# Engagement of DNA and H3K27me3 by the CBX8 chromodomain drives chromatin association

**DOI:** 10.1093/nar/gky1290

**Published:** 2018-12-29

**Authors:** Katelyn E Connelly, Tyler M Weaver, Aktan Alpsoy, Brian X Gu, Catherine A Musselman, Emily C Dykhuizen

**Affiliations:** 1 Department of Medicinal Chemistry and Molecular Pharmacology, Purdue University, West Lafayette, IN 47907, USA; 2 Department of Biochemistry, University of Iowa, Iowa City, IA 52242, USA

## Abstract

Polycomb repressive complex 1 (PRC1) is critical for mediating gene repression during development and adult stem cell maintenance. Five CBX proteins, CBX2,4,6,7,8, form mutually exclusive PRC1 complexes and are thought to play a role in the association of PRC1 with chromatin. Specifically, the N-terminal chromodomain (CD) in the CBX proteins is thought to mediate specific targeting to methylated histones. For CBX8, however, the chromodomain has demonstrated weak affinity and specificity for methylated histones *in vitro*, leaving doubt as to its role in CBX8 chromatin association. Here, we investigate the function of the CBX8 CD *in vitro* and *in vivo*. We find that the CD is in fact a major driver of CBX8 chromatin association and determine that this is driven by both histone and previously unrecognized DNA binding activity. We characterize the structural basis of histone and DNA binding and determine how they integrate on multiple levels. Notably, we find that the chromatin environment is critical in determining the ultimate function of the CD in CBX8 association.

## INTRODUCTION

Eukaryotic DNA is packaged into the cell nucleus in the form of chromatin. At its most basic level, chromatin is made up of repeats of nucleosome particles, which consist of ∼147 base-pairs (bp) of DNA wrapped around an octamer of histones H2A, H2B, H3 and H4. The N-terminus of each histone (and the C-terminus of H2A) protrude from the nucleosome core and are collectively known as the histone tails. These tails can be heavily post-translationally modified, catalyzed by a group of proteins known as ‘writers’. The modification of histone tails can alter chromatin structure directly and/or recruit or regulate chromatin associated proteins contributing to transcriptional changes (1,2). These interactions are mediated through ‘reader’ domains, that can recognize specific modification states (3,4).

The Polycomb Group (PcG) proteins, which are critical for lineage specification and adult stem cell maintenance (5), form two distinct histone writer complexes: Polycomb Repressive Complex 1 and 2 (PRC1 and PRC2) (6–8). According to the canonical mechanism for Polycomb, PRC2 catalyzes the trimethylation of lysine 27 on histone H3 (H3K27me3) (6), and PRC1 binds this modification, where it monoubiquitinates H2AK119 and compacts chromatin to repress transcription (9–11). In *Drosophila melanogaster*, recognition of H3K27me3 by dPRC1 is mediated by a chromatin modifier organization (chromo) domain, in the Polycomb (dPc) subunit (7,12). There are five paralogous proteins to dPc in mammals, referred to as chromodomain-containing chromobox subunits (CBX2,4,6,7,8) (13), that form mutually exclusive PRC1 complexes (14). The mammalian CBX chromodomains (CDs) demonstrate lower affinity for methylated histone peptides than does the dPc CD, and not all display specificity for H3K27me3 over H3K9me3 histone peptides *in vitro* (7,15,16). Of the five CBX CDs, the CBX8 CD demonstrates the weakest histone peptide binding (K_d_>500 μM) in these studies and no measurable specificity for H3K27me3 peptides (15). Meanwhile, live cell imaging studies suggest that H3K27me3 is important for the chromatin association of CBX8 (17), making the mechanism by which the CD contributes to CBX8 chromatin association and histone mark specificity unclear. A greater understanding of the CBX8 CD is not only desirable for understanding the fundamental mechanism of PRC1 function, but also for deciphering whether the CD is a good therapeutic target (18). Specifically, CBX8 plays an oncogenic role in several cancers including breast cancer and acute myeloid leukemia (19–21) and is overexpressed in numerous others, such as glioblastoma multiforme (GBM) (22–24). Inhibition of CBX8 may be a viable path for treatment of these diseases, and the CD may be targetable with small molecule inhibitors (25).

Here, we demonstrate that CBX8 association with chromatin is largely driven by the CD. Notably, we find that this is mediated through a combination of H3K27me3 binding, and a previously unrecognized interaction with DNA. We investigate the structural basis of both of these interactions, defining the root of moderate specificity for H3K27me3, and how histone and DNA binding integrate on multiple levels. Notably, despite the fact that *in vitro* histone tail binding is weak, and that nucleosome association *in vitro* is driven by DNA binding, we find that both DNA and H3K27me3 binding contribute to robust chromatin association *in vivo*, highlighting that the chromatin context is critical in determining ultimate reader domain function. These studies not only provide novel insight into reader domain and PRC1 function but will pave the way for the development of CBX8 inhibitors.

## MATERIALS AND METHODS

### Plasmids and constructs

CBX8 chromodomain construct was a gift from Cheryl Arrowsmith (Addgene plasmid #62514 (15)). GST-CBX8 chromodomain (residues 8–61) was generated using Infusion^®^ (Clontech) and the pGSTag vector (a gift from Gerald Crabtree). WT CBX8 gene was obtained from the Mammalian Gene Consortium (ID#4121509). The ΔCD (aa 77–389) was generated. QuikChange (Stravagene) was used to generate full length CBX8 W32A mutant. Additional full length CBX8 mutant constructs (R19A; R20A; R22A; R19,20,22A;) were purchased from GenScript and amplified. All CBX8 genes were cloned into the tet-inducible conditional lentiviral vectors TetO-FUW (a gift from Rudolf Jaenisch Addgene plasmid # 20323 (26)) containing the N-terminal V5 epitope with Infusion^®^ (Clontech). Infusion reactions were transformed in Stbl3 Competent cells (Invitrogen), DNA was isolated, and precipitated with 2 volumes ethanol and 0.1 volume sodium acetate for lentiviral transduction. CBX8 and control CRISPR guide RNAs were cloned into px459 v2.0 vector (a gift from Feng Zhang, Addgene plasmid #62988 (27)). EZH2 guide RNAs were cloned into Lenti_sgRNA_EFS_GFP (a gift from Chris Vakoc, Addgene plasmid #65656 (28)). Guide RNA sequences: sgCBX8: GCATGGAATACCTCGTGAAA, sgControl: GTAGCGAACGTGTCCGGCGT sgEZH2: CTGGCACCATCTGACGTGGC.

### Protein expression and purification

The recombinant CBX8 CD was expressed in BL21 (DE3) (New England Biolabs) *Escherichia coli* cells. Cells were grown in LB-medium or M9-minimal media supplemented with ^15^N-NH_4_Cl or ^15^N-NH_4_Cl and ^13^C-glucose. For unlabeled protein, cells were grown shaking at 215 rpm at 37°C until an OD ∼1.0 was reached and induced with 1 mM IPTG for 16–18 h overnight. For isotopically-enriched protein, cells were grown in LB-medium until an OD ∼1.0, spun down at 4000 rpm for 10 min, and resuspended in M9-medium (4 l LB cells per 1 l M9) supplemented with either ^15^N-NH_4_Cl or ^15^N-NH_4_Cl/^13^C-glucose. The cells were allowed to recover in M9 media for 1 h shaking at 18°C and induced with 1.0 mM IPTG for 16–18 h overnight. Cells were subsequently collected by centrifugation at 6000 rpm for 20 min, frozen in N_2_(l) and stored at –80°C.

For purification, cells were resuspended in a buffer containing 100 mM NaCl, 25 mM Tris (pH 7.5) supplemented with DNase I and lysozyme. Cells were then lysed using an Emulsiflex homogenizer (Avestin) or by sonication, and lysate cleared by centrifugation at 15 000 rpm for 1 h at 4°C. The soluble supernatant was incubated with glutathione agarose resin (ThermoFisher Scientific) rotating at 4°C for 1 h. The GST-tagged CBX8 CD was washed extensively with a high salt buffer containing 1 M NaCl and 25 mM Tris (pH 7.5) before elution with a buffer containing 150 mM NaCl, 25 mM Tris (pH 7.5) and 50 mM reduced glutathione. The GST-CBX8 CD was concentrated using a 3000 MWCO centrifugation filter unit to 2 ml and cleaved with TEV protease for 3 h at 25°C. The cleaved CBX8 CD was further purified using a combination of cation-exchange and size exclusion chromatography (Superdex 75, GE Healthcare Life Sciences). For NMR studies, ^15^N-CBX8 CD and ^15^N/^13^C-CBX8 CD were used in a final NMR buffer containing 100 mM NaCl and 40 mM phosphate buffer (pH 6.8). For EMSAs, the unlabeled CBX8 CD was used in a final buffer containing 25 mM phosphate buffer (pH 6.8), 25 mM NaCl, 1 mM EDTA, 1 mM DTT.

### Histone peptides

The unmodified H3 (1–44) and H3K27C (23–34) peptides were synthesized by GenScript. The H3K9me3 (1–21), H3K27me3 (23–34), Biotinylated H3 (21–44) and H3K27me3 (21–44) peptides were obtained from AnaSpec. For NMR studies, peptides were resuspended in H_2_O to a final concentration of 20 mM and pH adjusted to ∼7.0 with NaOH. For peptide pulldown experiments, peptides were resuspended to a final concentration of 1 μg/μl in 10% dimethyl sulfoxide (DMSO). Ethylcysteine alkylation of H3K27C peptide was performed as previously described (29). Briefly, peptide (2 mg) was resuspended in 8 M guanidinium chloride, 1 M HEPES pH 7.5, 1 M DTT and incubated for 1 h at 37°C. Following incubation, (2-bromomethyl)-trimethylammonium bromide (20 mg) was added for 2 h incubation at 50°C in the dark. Reaction was quenched with BME and HPLC purified. Reaction success was confirmed by mass spectrometry. Expected mass:1174.6, identified mass at 1174.8.

### DNA oligonucleotides

The single stranded DNA oligonucleotides (5′-GCGTTTAAGCG-3′ and 5′ CGCTTAAACGC-3′) were obtained from Integrated DNA technologies. For annealing, oligonucleotides were resuspended in 100 mM NaCl and 40 mM phosphate buffer (pH 6.8), heated to 90°C for 5 min and cooled overnight. The annealed DNA oligonucleotides were further purified by S75 size-exclusion chromatography in a buffer containing 100 mM NaCl and 40 mM phosphate buffer (pH 6.8) and concentrated to 3.2 mM in a 3000 MWCO centrifugal concentrator.

### Nucleosome reconstitution

Unmodified human histones H2A.1, H2B.1, H3.2 and H4 (T71C) were transformed into Rosetta2 (DE3) or BL21 (DE3) (New England Biolabs) and expressed in LB media. Cells were induced at OD ∼0.4 with 0.2 mM IPTG for histone H4 or 0.4 mM IPTG for histones H2A, H2B, and H3 for 3–4 h. The histones were extracted from inclusion bodies and purified by anion and cation exchange chromatography) (30).

Unmodified NCPs octamers were made largely following the protocol in (30). In short, equimolar amounts of histones H2A, H2B, H3 and H4 were mixed in a buffer containing 6M Guanidine HCl, 20 mM Tris (pH 7.5) and 10 mM DTT then dialyzed multiple times into a buffer containing 2 M KCl, 20 mM Tris (pH 7.5), 1 mM EDTA and 5 mM β-ME. The unmodified octamers were further purified using size exclusion chromatography (sephacryl S-200, GE Healthcare Life Sciences).

Unmodified nucleosome core particles (NCPs) were reconstituted using the unmodified octamers and the Widom 601 DNA (147 bp) through a desalting method (30). Unmodified octamers and the Widom 601 DNA (147 bp) were combined in equimolar ratios and desalted using a linear salt gradient from 2 M KCl to 150 mM KCl over 48 h. After reconstitution, NCPs were heat shocked for 30 min at 37°C for homogenous positioning on the Widom 601 DNA (147 bp). The NCPs were further purified using a 10–40% sucrose gradient. The purity and proper formation of unmodified NCPs was confirmed using native polyacrylamide gel electrophoresis.

### NMR spectroscopy

For assignment of backbone amide resonances, HNCACB and HN(CO)CACB experiments were collected on a 0.275 mM ^15^N/^13^C-CBX8 CD sample on a Bruker Avance II 500 MHz spectrometer with a 5mm triple resonance probe at 25°C. The triple resonance experiments were processed in NMRpipe (31) and further analysis carried out using CcpNmr (32). Initial backbone amide resonance assignments were generated using the PINE server (33) and further curated using CcpNmr (32).

Titrations experiments were performed by collecting ^15^N-HSQC spectra on 0.05–0.10 mM ^15^N-CBX8 CD in the apo state and upon addition of increasing molar ratios of the respective ligands. Titrations experiments were performed at 25°C (with the exception of H3K_c_27me3 that was performed at 16°C) on a Bruker Avance II 800 MHz NMR spectrometer equipped with a 5 mm triple resonance cryoprobe or a Unity Inova 600 MHz Oxford AS600 spectrometer equipped with a 5 mm triple resonance probe. The data was processed using NMRPipe (31) and further analysis performed using CcpNmr (32). To determine dissociation constants (*K*_d_), GraphPad PRISM was used for nonlinear least-squares analysis and the data fit to a single-site binding model accounting for ligand depletion using the equation:

\begin{equation*}\begin{array}{@{}*{1}{l}@{}} {\Delta \delta \ = \Delta {\delta _{{\rm max}}}\left( {\left( {\left[ {\rm L} \right] + \left[ {\rm P} \right] + {K_{\rm d}}} \right) } \right.}\\ {\quad \quad \left. { - \sqrt {{{\left( {\left[ {\rm L} \right] + \left[ {\rm P} \right] + {K_{\rm d}}} \right)}^2} - 4\left[ {\rm P} \right]\left[ {\rm L} \right]} } \right)/\left( {2\left[ {\rm P} \right]} \right)} \end{array}\end{equation*}

where [P] is the concentration of the CBX8 CD, [L] is the concentration of ligand, Δ*δ* is the normalized chemical shift change and Δ*δ*_max_ is the normalized chemical shift change at saturation, calculated as:

\begin{equation*}\Delta \delta \ = \sqrt {{{\left( {\Delta {\delta _{\rm H}}} \right)}^2} + {{\left( {0.20\Delta {\delta _{\rm N}}} \right)}^2}} \ \end{equation*}

where Δ*δ* is the chemical shift in parts per million (ppm).

Global *K*_d_ values were determined by averaging the individual *K*_d_ values for all resonances significantly perturbed in the titration experiments and reported as the average and standard deviation. A resonance was considered significantly perturbed if the Δ*δ* value was greater than the average plus one standard deviation after trimming 10% of residues (five resonances) with the largest Δ*δ* value. Individual resonances with *K*_d_ values greater than two standard deviations from the mean of the global *K*_d_ were removed from the analysis. If 50% or more of the individual residue *K*_d_ values were greater than two standard deviations from the global mean *K*_d_, than the global *K*_d_ was reported as lower limit (e.g. >0.8 mM).

### Cell culture

HEK293T cells were cultured in Dubecco's Modified Essential Media (DMEM), 10% fetal bovine serum (FBS, JR Scientific), 1% glutagro (Corning), 1% penicillin/streptomycin (Corning), 1% sodium pyruvate (Corning). GBM T98G cells were cultured in Eagle's Modified Essential Media, 10% FBS, 1% non-essential amino acids (Corning), 1% glutagro (Corning), 1% penicillin/streptomycin (Corning) and 1% sodium pyruvate (Corning). Hs68 cells were cultured in Dubecco's Modified Essential Media, 10% fetal bovine serum (JR Scientific), 1% penicillin/streptomycin (Corning) and 1% glutagro (Corning). All cells were grown at 37°C and 5% CO_2_. For generation of CBX8 and control CRISPR knockout lines, 200 000 T98G cells were plated in six-well 24 h prior to transfection. The respective vector (3.3 μg) was co-transfected with 13 μl of Fugene 6 (Promega). Media was changed 24 h post-transfection. Transfected cells underwent puromycin selection (2 μg/ml) for 3 days, 48 h post-transfection. EZH2 knockout lines were transduced with the MSCV_Cas9_puro vector (a gift from Chris Vakoc, Addgene plasmid #65655) (28) followed by the guide RNA vector.

### Lentiviral transduction

HEK293T cells were co-transfected with either TetOFUW constructs or pLenti CMV rtTA3 Hygro (w785-1) (a gift from Eric Campeau Addgene plasmid # 26730) and viral packaging vectors (pMD2.G and psPAX2). Viral supernatant was harvested and concentrated by ultracentrifugation at 17 300 rpm for 2 h, 72 h following transfection. Virus was resuspended in PBS and TetOFUW and rtTA were co-added to T98G and Hs68 cells and spun at room temperature for 1 h at 200 × g. Forty-eight hours after infection, cells were selected with puromycin (2 μg/ml) and hygromycin (200 μg/ml) for a week to generate stable cell lines. The doxycycline was added every 48 h at 1 μg/ml to induce expression.

### Sequential salt extraction

Protocol was performed as previously described (34). Briefly, 5 million cells were harvested, washed with PBS and lysed in buffer A (25 mM HEPES pH 7.6, 5 mM MgCl_2_, 25 mM KCl, 0.05 mM EDTA, 10% glycerol, 0.1% NP40) with protease inhibitor cocktail for 10 min at 4°C. Nuclei were pelleted at 5500 × g for 5 min at 4°C. The nuclei pellet was resuspended in 500 μl of mRIPA 0 mM NaCl (50 mM Tris pH 8.0, 1% NP40, 0.25% sodium deoxycholate) and incubated on ice for ∼10 min. Chromatin was pelleted at 6000 × g for 5 min at 4°C. Supernatant was collected, and the pellet was resuspended in mRIPA with 100 mM NaCl. This was repeated up to 500 mM NaCl. 4× SDS loading dye with beta-mercaptoethanol (BME) and immunoblot was performed. Immunoblots were quantitated using ImageJ (35) to calculate the percentage of CBX8 in each fraction, and two-tailed Student's *t*-tests were performed for each salt extraction comparison (*P < 0.05, **P < 0.01, ***P < 0.001, ****P < 0.0001) using PRISM GraphPad. For the EZH2i SSEs, cells were treated for 48 h with 1 μM GSK343 (Caymen Chemical) or DMSO prior to being harvested.

### Immunoprecipitation

T98G cell lines were harvested and washed with PBS. Cell membranes were lysed with buffer A with protease inhibitor cocktail for 15 min on ice. Nuclei were pelleted at 1000 × g for 5 min at 4°C. The nuclei pellets were resuspended in IP buffer (25 mM tris, 300 mM NaCl, 1 mM EDTA, 1% NP-40) and lysed on ice for 20 min. Chromatin was pelleted and supernatant was collected. Lysate was pre-cleared with 10 μl of Pierce™ Protein A/G Magnetic beads (ThermoFisher) overnight. Two hundred micrograms (200 μg) of lysate was incubated with 2 μg of V5 antibody (mouse, Invitrogen) for 2 h at 4°C. Following, 10 μl of Pierce™ Protein A/G Magnetic beads equilibrated in IP buffer were added for 1 h. IPs were washed 3× with IP buffer and resuspended in 4× SDS loading dye with BME and immunoblot was performed.

### Peptide pulldowns

T98G mutant cell lines were washed with PBS and harvested with Buffer A. Cells were incubated in Buffer A for 15 min on ice. Nuclei were pelleted at 1000 × g for 5 min at 4°C. The nuclei were re-suspended in IP buffer and lysed for 20 min on ice. Chromatin was pelleted and supernatant was collected. Biotinylated H3 (21–44) and H3K27me3 (21–44) peptides (2 μg, Anaspec) were pre-bound to streptavidin agarose resin (TriLink) for 1 h at 4°C. Following incubation, lysate (125 μg for WT, R19A, R20A, R22A or 375 μg for 3× RA and W32A) was added and incubated for 2 h at 4°C in peptide pulldown buffer (50 mM Tris pH 6.6, 150 mM NaCl, 1% NP-40, 0.5 mM DTT). Pulldowns were washed three times for 5 min with peptide pulldown buffer. Samples were resuspended in 4× SDS loading dye with BME and immunoblot was performed.

### Immunoblot and antibodies

Lysates were boiled and loaded on a 4–12% SDS-page gel (Invitrogen). Gels were transferred to PDVF membranes (Millipore) and incubated in 5% bovine serum albumin (BSA) in PBS-t (PBS with 0.1% Tween-20) prior to primary antibody. Blots were incubated at 4°C overnight in primary antibody. Blots were washed with PBS-t and incubated for an hour at room temperature in goat anti-rabbit or mouse conjugated to IRDye^®^ 800CW or IRDye^®^ 680 (LI-COR) secondary antibody. Blots were imaged on the Licor Odyessy^®^. Primary antibodies used: CBX8 (rabbit, 1:1000, Bethyl), V5 (mouse, 1:5000, Invitrogen), V5 (rabbit, 1:1000, Cell Signaling Technologies), EZH2 (mouse, 1:1000, BioRad), H3 (rabbit, 1:5000, Active Motif), H3K27me3 (mouse, 1:5000, Epigenetik), CBX4 (rabbit, 1:500, Bethyl), CBX7 (rabbit, 1:1000, Bethyl), CBX2 (mouse, 1:400, Santa Cruz).

### Electrophoretic mobility shift assays

Linear 601 DNA or reconstituted biotinylated mononucleosomes (Epicypher®) were incubated with CBX8 chromodomain in EMSA buffer (25 mM phosphate buffer pH 6.8, 25 mM NaCl, 1 mM EDTA, 1 mM DTT) at varying molar ratios for 15 min on ice. Samples (10 μl) containing 0.5 pmol nucleosome/0.25 pmol DNA, varying concentrations of chromodomain and 2× sucrose loading dye were loaded onto a prewarmed 6% (59:1) non-denaturing acrylamide gel and run in 0.4× TBE (pH 8.3) at 100 V on ice for 1 h. Gels were stained with ethidium bromide for 5 min and imaged on BioRad ChemiDoc™.

### RNA-Seq

T98G CBX8 KO cells with empty TetOFUW-empty and FUW-CBX8 were plated at a density of 80 000 cells per well of a six-well plate. Cells were harvested with Trizol^®^ (Invitrogen) and RNA was isolated according to manufacturer's instructions. Isolated RNA was further purified using the RNeasy^®^ kit (Qiagen). Library construction (100 bp, paired-end) and sequencing were carried out by Beijing Genomics Institute (BGI). The total RNA samples were enriched for mRNA by targeting polyadenylated (poly(A)) using oligo (dT) magnetic beads. Isolated mRNA was resuspended in fragmentation buffer and sonicated into short fragments of ∼200 bp. mRNA was reverse transcribed into a single strand of causing random hexamer-primers. The second strand of cDNA was synthesized using DNA polymerase and the double stranded cDNA was purified with magnetic beads. End reparation and 3′-end Adenine addition were performed subsequently. Thereafter, sequencing adaptors were ligated to the fragments and the fragments were enriched by PCR amplification. Finally, the library products were sequenced on the BGISeq500. The quality of the reads was evaluated with FastQC. Adapters were trimmed using Trimmomatic via the Galaxy web interface (36). Paired end reads were aligned to the Hg38 genome using HiSat2 on the Galaxy web interface (36). EdgeR was used to identify differentially expressed genes (FDR < 0.05) (37,38).

### qRT-PCR

Cells were plated at a density of 187 000 cells in 60 mm tissue culture plates and treated with 1 μg/ml of doxycycline for 72 h. Cells were harvested with Trizol^®^ (Invitrogen) and RNA was isolated according to manufacturer's instructions. Isolated RNA was further purified using the RNeasy^®^ kit (Qiagen). Two micrograms (2 μg) of isolated RNA was converted to cDNA using Verso™ cDNA synthesis kit (ThermoFisher). cDNA was diluted ∼4-fold with water and qRT-PCR was performed with maxima SYBR green master mix (Thermo) and run on the BioRad CFX Connect™ thermo cycler. Six biological replicates were performed in technical triplicate. Data was normalized to the primer efficiency and *B2M* expression (39).

**Table:** 

Gene name	Forward primer (5′-3′)	Reverse primer (5′-3′)
*A2M* (40)	CGGAGAATGACGTACTCCACT	TGGGTTGGTCCTTTCACTTGG
*ALDH1A3* (40)	ACCTCTCACCGCCCTTTATCT	GTGAAGGCGATCTTGTTGATCT
*SLPI* (40)	GAGATGTTGTCCTGACACTTGTG	AGGCTTCCTCCTTGTTGGGT
*CTSF* (40)	CCCTCCAATGCCTACTCGG	CCAGCTTCTGCTCGTTCTG
*VAV3* (41)	ACAAGGAGCCAGAACATTCAG	TTGCACAGAAGTCATACCGAG
*B2M*	TGCTGTCTCCATGTTTGATGTATCT	TCTCTGCTCCCCACCTCTAAGT

### Chromatin immunoprecipitation

Two million Hs68 cells were harvested from 10 cm plates and washed with PBS. The cells were re-suspended in CiA Fix buffer (50 mM HEPES pH 8.0, 1 mM EDTA, 0.5 mM ETGA, 100 mM NaCl) and crosslinked with 1% formaldehyde for 10 min at room temperature. Crosslinking was quenched with 0.125 M glycine for 5 min at 4°C. Following, cells were washed once with PBS then resuspended in CiA NP Rinse buffer 1 (50 mM HEPES pH 8.0, 140 mM NaCl, 1 mM EDTA, 10% glycerol, 0.5% NP-40, 0.25% Triton X) for 10 min. Cells were pelleted at 1200 × g for 5 min at 4°C. The supernatant was removed and the cells were resuspended in CiA NP rinse buffer 2 (10 mM Tris pH 8.0, 1 mM EDTA, 0.5 mM EGTA, 200 mM NaCl). Cells were collected by centrifugation, 1200 × g for 5 min. Supernatant was removed and the cells were washed twice with shearing buffer (0.1% SDS, 1 mM EDTA, 10 mM Tris–HCl, pH 8.0). Following, cells were resuspended in shearing buffer and sonicated for 7 min with the probe (Branson 250). Lysate was centrifuged at 21 000 × g for 15 min to remove debris. Supernatant was collected and pre-cleared overnight with Pierce™ Protein A/G magnetic beads (ThermoFisher). For immunoprecipitation, cell lysate was divided in half and incubated with 1 μg of antibody and Pierce™ Protein A/G magnetic beads for 3 h. Ten percent (10%) input was saved for downstream analysis. The IPs were washed two times for 3 min at RT with IP buffer (50 mM HEPES/KOH pH 7.5, 300 mM NaCl, 1 mM EDTA, 1% Triton X, 0.1% DOC, 0.1% SDS) followed by a DOC (10 mM Tris pH 8.0, 0.25M LiCl, 0.5% NP-40, 0.5% dexoycholate (DOC), 1 mM EDTA) wash and a 1× TE wash. Protein was eluted from beads (1% SDS, 0.1 M NaHCO_3_) two times for 20 min at RT. Samples, including input, were treated with RNase A at 37°C for 30 min followed by proteinase K for 3 h at 55°C. Samples were un-crosslinked overnight at 65°C. Phenol chloroform followed by isopropanol precipitation were used to extract and isolate the DNA. DNA was resuspended in 20 μl TE for qPCR analysis. Antibodies used for IP were V5 (Invitrogen, mouse), CBX8 (Bethyl, rabbit), IgG (Santa Cruz, mouse; CST, rabbit). qPCR was performed on the isolated ChIP DNA. qPCR was performed using maxima SYBR green master mix (Thermo) and run on the BioRad CFX Connect™ thermo cycler. Three biological replicates were performed in technical triplicate. Enrichment was determined by percent input.

**Table:** 

Gene name	Forward primer (5′-3′)	Reverse primer (5′-3′)
*LMNB2*	CCGAATCTCTGAAATGAAAGTCCATGC	TTAAAGATCTGAGGGACTCCTCAGTC
*CCND2* (42)	ACTGTCTGAAATGAAGGTGAAGC	GATTTGATGGACACTTGGTTTGT
*GATA6* (42)	GCCTCTCCATTCCAGAGTTTT	TCCAGAAACCGTTCTCATCC
*RUNX3* (42)	TCAAAAGGCATCCGCCTCTCCGT	AAGGATGCACCTGCCGGGAATTG

### Genome-wide data analysis

Published annotated datasets were downloaded from Encyclopedia of DNA Elements (ENCODE) or Gene Expression Omnibus (GEO) as BED files (43,44). Peaks for CBX8, H3K4me3, H3K9me3, H3K27me3 and DNAse hypersensitivity were called in reference to GrCh38. All data sets were imported into R Studio. Peak overlaps were determined using the ChIPpeakAnno package (45,46). Overlaps were defined as being within 150 base pairs of each other and on the same strand. Accession numbers for CBX8: GSM830987, GSM1295078, GSM1295089, ENCFF001SYX; H3K27me3: GSM1295084, GSM1295094, ENCFF001SZF; H3K4me3: GSM1295085, GSM1295095, ENCFF001SZJ; H3K9me3: ENCFF001SZN; DNase-seq: ENCFF100IJK.

## RESULTS

### CBX8 associates robustly with chromatin *in vivo* through its chromodomain

**Figure 1.**
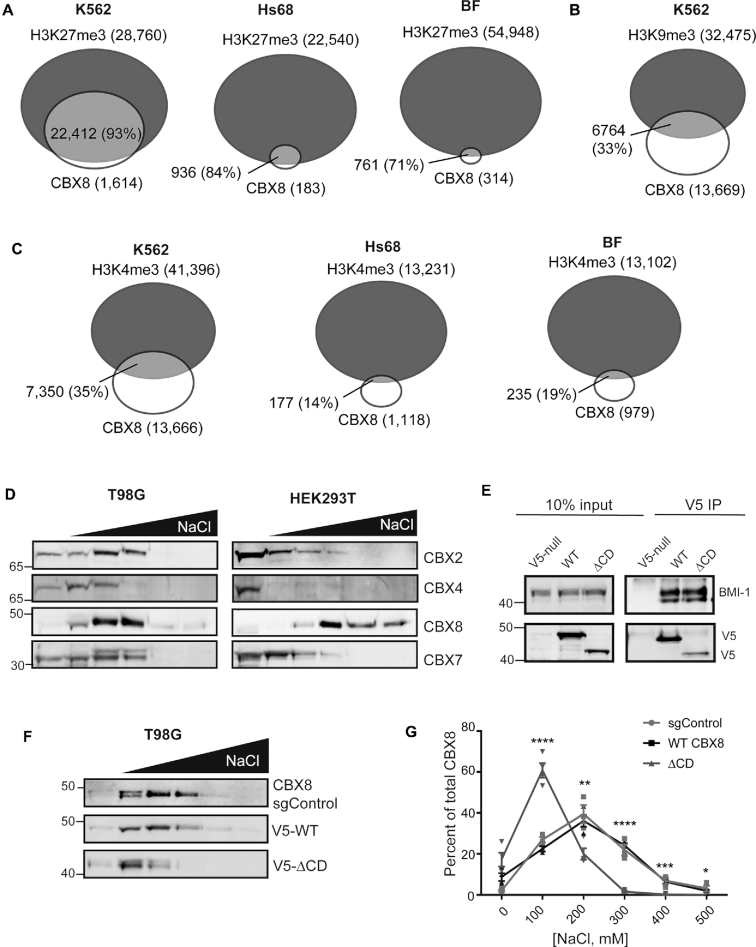
CBX8 robustly associates with chromatin via its chromodomain. (**A**) Genome-wide analysis of CBX8 and H3K27me3 peak overlaps in K562, (47) Hs68, (42) and BF (42) cell lines, respectively. Numbers indicate number of called peaks, overlap percent of total CBX8 peaks. (**B**) Analysis of CBX8 and H3K9me3 overlaps in K562 cells. (**C**) CBX8 and H3K4me3 overlaps in K562, Hs68, and BF cells, respectively. (**D**) Sequential salt extraction in T98G (left) and HEK293T (right) cells examining endogenous CBX paralog chromatin association. Paralog elution patterns were detected using immunoblot and antibodies against the respective paralog. (**E**) Anti-V5 co-immunoprecipitation of WT and ΔCD CBX8, with detection of the PRC1 subunit BMI-1 to verify PRC1 complex incorporation. (**F**) Representative SSE of sgControl T98G cells (anti-CBX8), wild type and ΔCD re-expression (anti-V5) in T98G sgCBX8 cells. (**G**) Quantitation of SSEs displayed in (F), sgControl *n* = 3, WT and ΔCD *n* = 4 biological replicates, errors bars represent standard error of the mean (SEM), *P*-values were calculated using Student's two-tailed *t*-test: *P < 0.05, **P < 0.01,***P < 0.001, ****P < 0.0001

Previous studies have revealed that the CBX8 CD demonstrates very weak affinity *in vitro* for H3K27me3 and H3K9me3 peptides with indeterminate specificity (15). However, live cell imaging suggests that H3K27me3 is important for CBX8 chromatin targeting (17). To further assess chromatin specificity *in vivo*, we interrogated available CBX8 and corresponding histone modification genome-wide data sets to determine if CBX8 binding correlates to either H3K27me3 or H3K9me3. In the chronic myeloid leukemia cell line K562, 93% of the CBX8 peaks were found to overlap (within 150 base pairs) with H3K27me3 peaks (Figure 1A) (47). Additional analysis in the neonatal foreskin fibroblast cell line Hs68 and breast fibroblast cell line (BF) (42), revealed an 84% and 71% overlap, respectively (Figure 1A). In contrast, only 33% of CBX8 peaks correlate with the presence of H3K9me3 in the K562 cell line (Figure 1B) (47). There was a similarly low correlation between CBX8 peaks and the activating mark H3K4me3 in K562 cells (∼35%) as well as Hs68 and BF cells (∼15%) (Figure 1C). This suggests that CBX8 preferentially associates with H3K27me3 *in vivo*. It also suggests that since CBX8 interacts with only a subset of total H3K27me3 peaks, additional chromatin targeting mechanisms must be involved.

To further assess chromatin association of CBX8, we investigated its interaction with bulk chromatin using a sequential salt extraction (SSE) assay, in which relative binding is determined by the salt concentration required to elute proteins from chromatin (34). We initially compared the binding profile of CBX8 to several other CBX paralogs in two distinct cell lines, human embryonic kidney (HEK) 293T and GBM T98G. We observed that endogenous CBX8 from T98G cells associates with chromatin similarly to the other paralogs (Figure 1D, left). Interestingly, in HEK293T cells, CBX8 demonstrates a stronger chromatin association compared to CBX2, CBX4, and CBX7 (Figure 1D, right) suggesting there may be cell-type specific contributions to chromatin binding.

To evaluate the contribution of the CBX8 CD to this association, we deleted the corresponding amino acids 1–76 and re-expressed either this CD deletion (ΔCD) or wild-type (WT) CBX8 in a GBM T98G CBX8 knockout (sgCBX8) cell line using a doxycycline-inducible system. Importantly, both wild-type and ΔCD expressed CBX8 are incorporated normally into the PRC1 complex (Figure 1E). Using the SSE assay, we confirmed that WT CBX8 re-expression in T98G cells has a similar elution pattern to endogenous CBX8 (sgControl, Figure 1F); and that the ΔCD displays a reduction in bulk chromatin affinity compared to WT CBX8 (Figure 1F). In order to quantitatively assess binding differences, the amount of CBX8 in each fraction was assessed using ImageJ (35) and normalized to the total amount present (sum of all fractions), reported as the percentage of CBX8 eluting at each salt concentration. Quantitation revealed that the CD deletion significantly reduced the affinity of CBX8 for chromatin (Figure 1G) suggesting that the CD plays a critical role in CBX8 chromatin binding. Taken together, our data suggests that CBX8 specifies for H3K27me3 *in vivo* and that robust chromatin association is largely dependent on the CD.

### The CD preferentially recognizes H3K27me3 *in vitro*

**Figure 2.**
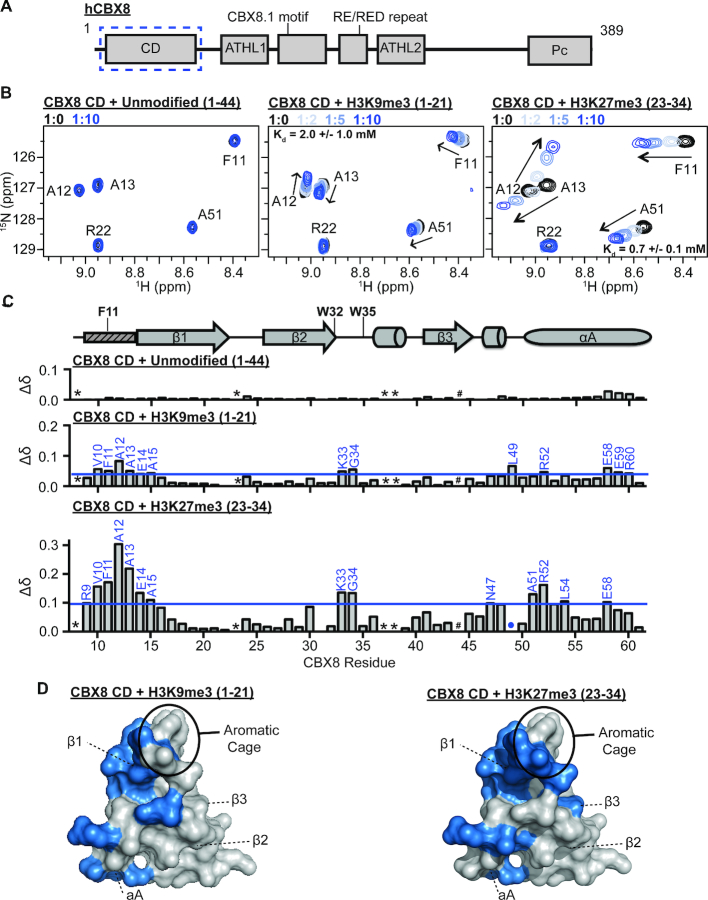
CD preferentially recognizes H3K27me3 *in vitro*. (**A**) Domain and motif architecture of the full-length CBX8 protein. The chromodomain used in this study is enclosed in a blue dotted box. (**B**) ^1^H–^15^N-HSQC overlays for ^15^N-CD upon addition of increasing concentrations of unmodified H3 (1–44, left), H3K9me3 (1–21, middle) or H3K27me3 (23-34, right) histone peptides. A selected region of each spectra is shown for clarity. Molar ratio of CD:peptide is color coded as shown in legend. (**C**) The normalized chemical shift perturbation (Δδ) between the apo and bound states (1:10 ratio) of CD resonances plotted against CBX8 residue number for each histone peptide tested. CSPs were considered significant if greater than one the mean plus one standard deviation and are labeled in blue. The secondary structure of CD from the crystal structure PDBID 3I91 is diagramed above the Δδ plots with the aromatic cage residues labeled. The small rectangle with dashed lines represents the region of CD that undergoes a conformational change between apo and histone bound states in the crystal structure. * indicates missing resonances, # indicates proline residue and blue dots represent resonances that broaden beyond detection during the experiment. D) Residues with significant CSPs upon addition of H3K9me3 (left) or H3K27me3 (right) highlighted in blue on a surface representation of the CD (PDB 3I91).

Previous *in vitro* studies demonstrated weak affinity of the CD for both H3K9me3 and H3K27me3 peptides. Because binding was beyond the limit of detection for the technique used, specificity was not distinguishable (15,16). To further assess the CD specificity, we utilized nuclear magnetic resonance (NMR) spectroscopy, a technique well suited to study weak binding interactions. An initial ^1^H–^15^N-heteronuclear single quantum coherence (HSQC) spectrum of ^15^N-CD (residues 8–61, Figure 2A) reveals 49 main chain resonances of the 53 expected assuming fast conformational exchange (Supplementary Figure S1A). Resonances are well dispersed in both ^1^H and ^15^N dimensions, indicating that the CD is well folded and amenable to NMR studies. All 49 backbone amide resonances were assigned using HNCACB and HN(CO)CACB triple-resonance experiments (Supplementary Figure S1A).

To investigate histone tail binding and CD specificity, sequential ^1^H-^15^N-HSQC spectra were collected on ^15^N-labeled CD upon addition of peptides corresponding to unmodified H3 (residues 1–44), H3K9me3 (residues 1–21), or H3K27me3 (residues 23–34). Addition of increasing concentrations of the H3K9me3 and H3K27me3 peptides resulted in significant chemical shift perturbations (CSPs) in the CD spectrum, suggesting an interaction with both peptides (Figure 2B and Supplementary Figure S2A). In contrast, addition of the unmodified H3 peptide resulted in no significant CSPs, indicating that binding is methylation dependent (Figure 2B, Supplementary Figure S2A). Dissociation constants (*K*_d_) were calculated for H3K9me3 and H3K27me3 by fitting normalized CSPs (Δ*δ*) to a one-site binding model accounting for ligand depletion (see methods for details). This yielded a *K*_d_ = 0.7 mM for H3K27me3 and *K*_d_ = 2.0 mM for H3K9me3 (Supplementary Figure S2B,C). Thus, though binding is weak to both modifications, the CD preferentially binds H3K27me3 *in vitro* (Supplementary Figure S2D).

To determine the structural basis of complex formation and specificity, CSPs were further assessed for both peptides. Plotting normalized CSPs between the apo and peptide-bound CD as a function of primary amino acid sequence shows that both H3K9me3 and H3K27me3 titrations resulted in CSPs at residues in the N-terminal portion of the β1 strand and the β2/β3 loop of the CD (Figure 2C). When plotted onto the crystal structure of the CD previously solved in complex with H3K9me3 (PDBID:3I91), these residues cluster in and around the aromatic cage consistent with the canonical histone binding pocket (Figure 2D, Supplementary Figure S4A,B), as well as in residues at the N-terminus of the αA helix (12,15). Notably, these primarily hydrophobic residues in the αA helix are substantially more perturbed upon binding H3K27me3 than H3K9me3, suggesting that these residues are important for specificity (Figure 2C). The two methylated lysines have significant sequence similarity in surrounding residues with sequences of AARKme3S (H3K27me3) and TARKme3S (H3K9me3). Notably, in the crystal structure of the CD bound to H3K9me3 (PDBID 3I91) (15), the polar H3T6 (–3 position) is in close proximity to these hydrophobic residues. Thus, it follows that the more hydrophobic alanine found at the -3 position of H3K27me3 (A24) would be preferred. Indeed, previous studies found mutating H3A24 to T24 results in significantly reduced affinity of the CBX8 CD for H3K27me3 (15). In addition, a recent peptide inhibitor screen towards the CD identified high affinity peptide mimetics that utilized bulky aromatic moieties at the –3 position (25).

The large CSPs around residues V10-A13 also support a conformational change seen in the previously published crystal structure. Specifically, the structure of the CD without H3K9me3, shows that these residues are disordered, resulting in an only partially formed aromatic cage with F11 positioned in the methyl-lysine binding pocket. In comparison, in the co-crystal structure of CBX8 bound to H3K9me3, V10-A13 are structured and form part of an extended β1 strand allowing for proper orientation of F11 and complete formation of the aromatic cage (15). Our NMR data is consistent with this conformational change.

Taken together, our data reveals that though the CD binds weakly it has specificity for H3K27me3 *in vitro*, consistent with the *in vivo* data from genome-wide associations.

### The CD association with nucleosomes is driven by interactions with DNA

**Figure 3.**
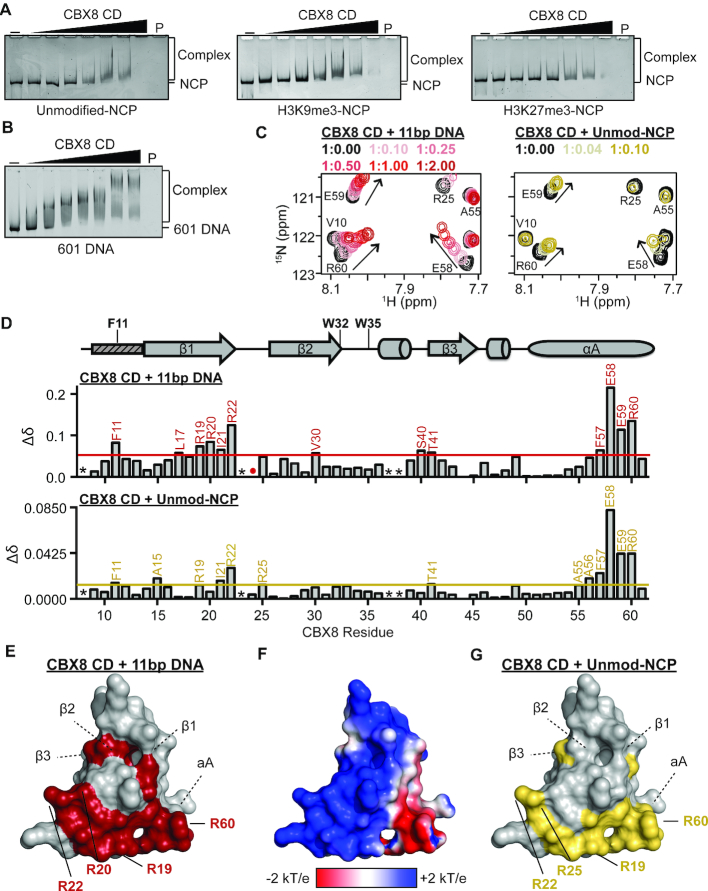
CD association with nucleosomes is driven by interactions with DNA through an arginine-rich basic patch. (**A**) EMSAs performed with CD and unmodified (left), H3K9me3 (middle) and H3K27me3 (right) NCPs. Shown are representative gels from a triplicate of experiments. (**B**) EMSAs performed with CD and the 147 bp 601 DNA. Shown is a representative gel from a triplicate of experiments. (**C**) ^1^H–^15^N-HSQC overlays for ^15^N-CD upon addition of increasing concentrations of an 11bp DNA (left) and unmodified NCP (right). Molar ratio of CD:DNA and CD:umodified NCP are color coded as shown in legend. A selected regions of the CD spectrum is shown for clarity. (**D**) Normalized CSP (Δδ) between the apo and DNA-bound (1:2.00 ratio) spectra are plotted against CBX8 residue number (top). Normalized CSP (Δ*δ*) between the apo and unmodified NCP-bound (1:0.10 ratio) spectra are plotted against CBX8 residue number (bottom). CSPs were considered significant if greater than the mean plus one standard deviation and are labeled in red or gold for 11 bp DNA and unmodified NCP, respectively. The secondary structure of CD from the crystal structure PDBID 3I91 is diagramed above the Δδ plot with the aromatic cage residues labeled. The small rectangle with dashed lines represents the region of CD that undergoes a conformational change between apo and histone bound states in the crystal structure. * indicates missing resonances, # indicates proline residue and red/gold dots represent resonances that broaden beyond detection during the experiment. (**E**) Residues with significant CSPs upon addition of the 11 bp DNA plotted onto a surface representation of the CD (PDBID 3I91) and colored red. Arginine residues that form the basic patch are shown as sticks. (**F**) APBS surface electrostatic representation of the CD (PDBID 3I91). (**G**) Residues with significant CSPs upon addition of the unmodified NCP plotted onto a surface representation of the CD (PDBID 3I91) and colored gold. Arginine residues that form the basic patch are shown as sticks.

Our NMR analysis reveals that the CD preferentially binds H3K27me3 *in vitro*; however, the generally weak affinity for histone tail peptides *in vitro* is inconsistent with this driving robust chromatin association. In order to determine if the CD makes additional contacts outside of the histone tail, we investigated binding in the context of methylated nucleosomes. Notably, a commonly utilized amino-ethylcysteine methyl-lysine analog (MLA) was not bound by the CD as has been seen with a subset of other reader domains (Supplementary Figure S2A) (48,49). Thus, electrophoretic mobility shift assays (EMSAs) were performed with nucleosome core particles (NCPs) reconstituted with the 147bp 601 DNA and unmodified H3 or methylated H3 (H3K9me3 or H3K27me3) generated by native chemical ligation (purchased from Epicypher). The addition of increasing concentrations of the CD resulted in significant changes in the mobility of all NCPs tested (Figure 3A, Supplementary Figure S3A), independent of methylation status. Notably, NCPs containing H3K9me3 and H3K27me3 resulted in changes in mobility at similar concentrations as the unmodified NCP, suggesting that robust association by the CD is driven primarily by interactions outside of the histone methyl mark. EMSAs of the CD with 601 DNA alone also indicated a robust interaction (Figure 3B, Supplementary Figure S3B). Together, this suggests that NCP binding by CD is driven primarily by interactions with DNA.

### The CD binds linear and nucleosomal DNA through an arginine-rich basic patch

In order to further characterize the DNA binding by the CD, we performed NMR titration experiments with an 11 bp DNA segment. Addition of increasing concentrations of unlabeled 11 bp DNA into ^15^N-CD resulted in significant CSPs for a subset of CD resonances (Figure 3C, Supplementary Figure S3c, d). Interestingly, some resonances with CSPs demonstrated curvature, suggesting binding that is more complex than a simple one-to-one complex. This makes the calculation of *K*_d_ values from the CSPs difficult, however as compared to the titration with H3K27me3 in which saturation is reached at 1:7 molar ratio, saturation with DNA is reached at ∼1:1 molar ratio suggesting that the CD binds DNA with a substantially higher affinity than H3K27me3, likely with a *K*_d_ <10 μM.

To determine the structural basis for interaction with DNA, CSPs between the free and DNA-bound states were assessed as a function of CD sequence. Residues with significant CSPs localized to the C-terminal portion of the β1 strand and the αA helix (Figure 3D). Mapping the residues with significant CSPs onto the structure of the CD revealed that DNA binding is mediated through a cluster of arginine residues (R19, R20, R22 and R60) that form a basic patch on the opposite face of the histone binding pocket (Figure 3E,F, Supplementary Figure S4C).

To determine if the CD interaction with unmodified nucleosomes is indeed being mediated through DNA binding as suggested in the EMSAs, an NMR titration was performed using recombinant unlabeled, unmodified NCPs (reconstituted with the 147 bp 601 sequence). Addition of increasing concentrations of unmodified NCPs resulted in significant CSPs in a subset of residues indicating binding, accompanied by global intensity decreases, which are expected due to the size of the complex (Figure 3C, Supplementary Figure S3D). Analysis of CSPs as a function of residue revealed an almost identical binding pocket as was observed with the 11 bp DNA (Figure 3D,G, Supplementary Figure S4D), and notably the CSPs followed nearly identical trajectories (Figure 3C) suggesting a similar binding mechanism. Together this confirms that NCP binding is driven through contacts with DNA, mediated by a basic patch on the CD surface.

### The CD can interact with DNA and H3K27me3 simultaneously

**Figure 4.**
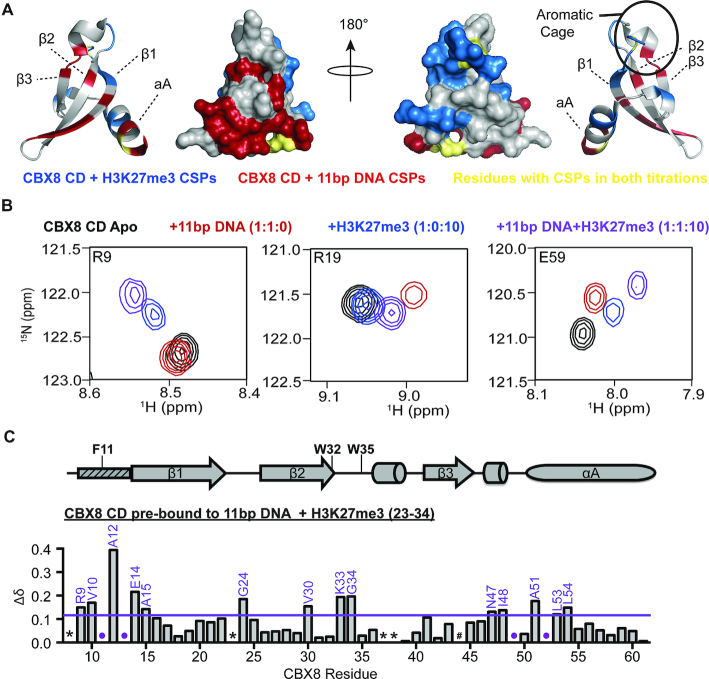
CD can interact with DNA and H3K27me3 simultaneously. (**A**) Normalized CSP (Δδ) between the apo and DNA-bound state (red) and apo and H3K27me3-bound state (blue) plotted onto a cartoon and surface representation of the CD to show largely non-overlapping binding sites. Residues perturbed in both titration experiments (F11 and E59) are colored gold. Two selected views are shown that are rotated 180° about the y-axis. (**B**) ^1^H–^15^N-HSQC overlays for ^15^N-CD in the apo (black, 1:0:0 ratio), bound to an 11bp DNA (red, 1:1:0 ratio), bound to H3K27me3 (blue, 1:0:10), or bound to both 11 bp DNA and H3K27me3 (purple, 1:1:10 ratio). Shown are resonances for selected residues in the histone binding pocket (R9, left), DNA binding pocket (R19, middle) and a residue sensitive to both DNA and histone binding (E59, right) are shown. (**C**) normalized CSP (Δδ) between the DNA-bound (1:1:0) and DNA and H3K27me3 bound (1:1:10 ratio) plotted against CBX8 residue number. CSPs were considered significant if greater than the mean plus one standard deviation and are labeled in purple. The secondary structure of CD from the crystal structure PDBID 3I91 is diagramed above the Δδ plot with the aromatic cage residues labeled. The small rectangle with dashed lines represents the region of CD that undergoes a conformational change between apo and histone bound states in the crystal structure. * indicates missing resonances, # indicates proline residue and purple dots represent resonances that broaden beyond detection during the experiment.

Comparison of CSPs for the CD upon binding to histone peptide or DNA revealed largely non-overlapping binding sites for DNA and H3K27me3, except for residues F11 and E59, which show significant CSPs upon binding either ligand (Figure 4A, Supplementary Figure S5A). This suggests that both may bind contemporaneously. To test this, NMR titrations were performed in which increasing concentrations of H3K27me3 peptide was added to ^15^N-CD pre-bound to the 11 bp DNA. This resulted in significant CSPs indicating that the DNA-bound CD can interact with the H3K27me3 peptide (Figure 4B, compare red and purple spectra). Comparison of spectra for the CD in the presence of H3K27me3 alone, DNA alone, or both H3K27me3 and DNA revealed unique chemical shift values for most CD residues (Figure 4B, Supplementary Figure S6C). This is consistent with the formation of a ternary complex but notably suggests that the bound state is unique as compared to either binary complexes. Residues with significant CSPs upon addition of H3K27me3 (23–34) to the CD pre-bound with DNA largely mapped to the determined histone binding pocket (Figure 4C, Supplementary Figure S5B); however, smaller CSPs were also seen in the DNA binding pocket. Notably, these CSPs are on a trajectory toward a state unique from the apo, DNA, or peptide bound states, suggesting that the DNA and histone binding are not completely independent of each other (Figure 4B middle, Figure 4C). Consistent with this finding, analysis of the CSPs for titration of H3K27me3 into the DNA-bound CD revealed an affinity of *K*_d_ = 0.2 mM, ∼3× tighter than that observed to the CD alone (Supplementary Figure S6E).

This increase in affinity may in part be due to an increased local concentration of peptide, as histone tails are known to bind to DNA (50–53); however, it may also be due to DNA mediated stabilization of the methyl-lysine binding pocket. As mentioned above, the CD aromatic cage is ill-formed in the absence of histone peptide, with residue F11 pointing into the aromatic cage, which must rearrange upon binding the methylated lysine. F11 is one of two residues observed to have significant CSPs upon binding of either DNA or H3K27me3, and notably, the perturbation upon DNA binding follows a similar trajectory to that seen upon titration of H3K27me3. This suggests that DNA binding may stabilize the aromatic cage, increasing the affinity for peptide. Importantly, however, a ternary complex and increase in affinity are also seen with H3K9me3, indicating that this does not lead to increased specificity for methyl marks (Supplementary Figures S5B, S6A,B,D). Taken together, our data indicated the CD can simultaneously bind DNA and H3K27me3 and that DNA binding enhances methyl-lysine binding.

### The CD interaction with DNA and H3K27me3 are important for CBX8 chromatin association

**Figure 5.**
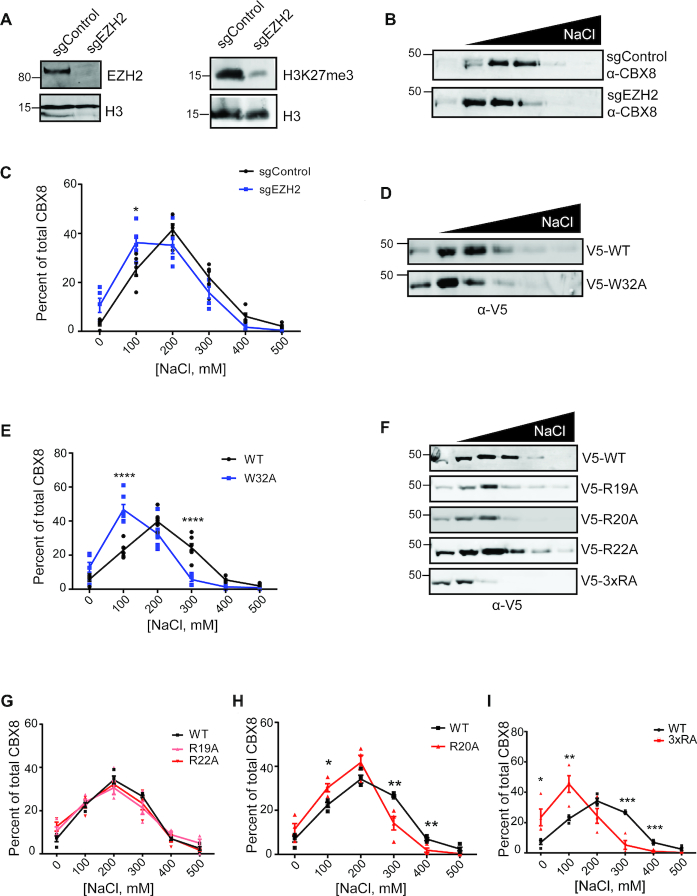
Methyllysine and DNA binding contribute to CBX8 chromatin association. (**A**) Immunoblots of sgEZH2 KO and sgControl cell lysate demonstrating EZH2 knockout efficiency (anti-EZH2, anti-H3 loading control) (left) and reduced H3K27me3 levels (anti-H3K27me3, anti-H3 loading control) (right). (**B**) Representative SSE of endogenous CBX8 in T98G sgEZH2 and sgControl cells (anti-CBX8). (**C**) Quantitation of CBX8 eluted in each fraction as a percent of total CBX8, *n* = 5 biological replicates, blue denotes sgEZH2 cell line, black denotes sgControl cell line. (**D**) Representative immunoblot of W32A SSE (anti-V5) in T98G sgCBX8 cells. (**E**) Quantitation of amount of CBX8 in each fraction as a percent of total CBX8, *n* = 4, 7 biological replicates for WT and W32A, respectively. WT is denoted in black and W32A is denoted in blue. (**F**) Representative SSE of DNA binding mutants (anti-V5) in T98G sgCBX8 cells. (**G–I**) Quantitation of amount of CBX8 in each fraction as percent of total CBX8, *n* = 4 biological replicates for each mutant compared to WT. DNA mutants are denoted in red (R19A, light red), WT is denoted in black. For all quantitation, error bars represent SEM, *P*-values were calculated using two-tailed Student's *t*-test, *P < 0.05, **P < 0.01, ***P < 0.001, ****P < 0.0001.

To investigate the importance of the CD DNA and histone binding in CBX8 chromatin association *in vivo*, SSE assays were carried out upon disrupting either the histone or DNA interaction of the CD. SSE is a way to measure bulk chromatin binding properties of a protein, and as such, the disruption of essential chromatin binding domains results in small, but highly reproducible, shifts in elution patterns (34,54). To initially assess the importance of H3K27me3 binding on CBX8 chromatin association, we generated a stable EZH2 knockout (sgEZH2) in T98G cells to reduce global H3K27me3 levels (Figure 5A) and performed sequential salt extractions on CBX8. Upon knockout of EZH2, we observed a decrease in the affinity of CBX8 for bulk chromatin compared to the sgControl cell line (Figure 5B). Quantification of the salt extractions indicates a significant reduction in the affinity of CBX8 for chromatin upon loss of H3K27me3 (Figure 5C). We additionally performed SSE with the WT CBX8 re-expression cells treated for 48 hours with the EZH2 inhibitor (EZH2i) GSK343 (Supplementary Figure S7A). In the presence of GSK343, we observed a significant decrease in the affinity of CBX8 for chromatin (Supplementary Figure S5B) that mimics the EZH2 knockout (Supplementary Figure S7C).

To further examine the role of methyl-lysine binding in CBX8 chromatin association, we generated a previously published aromatic cage mutation, W32A, that disrupts CBX chromatin localization (16,55). The V5-tagged W32A mutant was re-expressed in the T98G CBX8 knockout cell line and incorporated into the PRC1 complex (Supplementary Figure S7D). Peptide pulldowns confirm that W32A does not bind H3K27me3 (Supplementary Figure S7E). SSE with the V5-W32A expressing cells demonstrated a significant reduction in CBX8 chromatin binding (Figure 5D, E). When compared to the binding profile of CBX8 in sgEZH2 cells, we observe a similar elution pattern albeit more pronounced likely due to the incomplete H3K27me3 depletion in the sgEZH2 cells (Supplementary Figure S7F). Importantly, the W32A mutant did not reduce CBX8 chromatin affinity to the same severity of the ΔCD (Supplementary Figure S7G). Together this data shows that despite the weak affinity of the CD for H3K27me3 in the context of peptides and mono-nucleosomes, this interaction is indeed important for CBX8 chromatin association. These results also indicate that chromatin binding is not completely abrogated in the absence of methyl-lysine binding, suggesting that H3K27me3 is not the only contributing factor in CBX8 association with chromatin.

To assess the importance of the CD-DNA interaction in CBX8 chromatin association *in vivo*, we mutated residues identified as important for binding in the NMR studies. Specifically, R19, R20, and R22 were mutated to alanine individually (R19A, R20A, R22A) or together (3×RA). The V5-tagged mutants were re-expressed in the T98G CBX8 knockout cell line earlier described and incorporated into the PRC1 complex as observed by immunoprecipitation (Supplementary Figure S7D). As expected, these mutants still engaged H3K27me3 in a peptide pulldown assay (Supplementary Figure S7E).

To examine the contribution to bulk chromatin binding affinity, SSE assays were carried out with all mutants (Figure 5F). No significant changes were observed in bulk chromatin binding for R19A or R22A (Figure 5G), however R20A led to a small but significant increase in the amount of CBX8 eluted at 100 mM NaCl compared to WT (Figure 5H). In comparison, the triple arginine mutant (3×RA) was consistently eluted at lower salt concentration as compared to WT (Figure 5I) indicating significantly weakened chromatin binding. Interestingly, when compared to the ΔCD SSE, there was not a significant difference in affinity for chromatin (Supplementary Figure S7H). This suggests that CBX8 chromatin binding is driven through DNA interactions.

**Figure 6.**
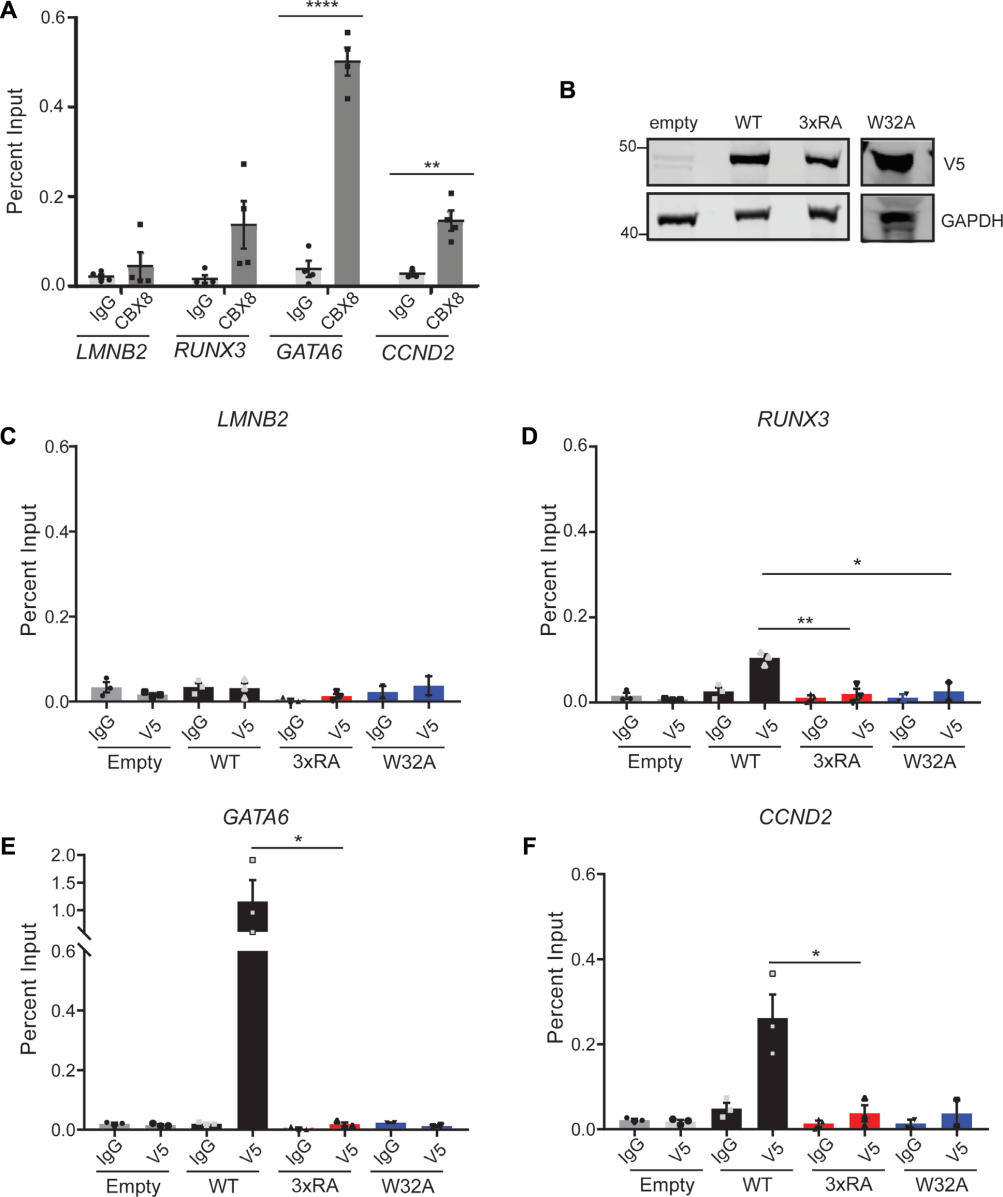
DNA and H3K27me3 binding are important for binding at discrete genomic loci. ( **A**) ChIP-qPCR of CBX8 at *LMNB2* (negative locus), *RUNX3, GATA6* and *CCND2n* = 4 biological replicates performed in technical triplicate. (**B**) Immunoblot analysis of V5 CBX8 WT, 3xRA and W32A expression in Hs68s as observed by α-V5 staining, GAPDH serves as a loading control. (**C–F**) ChIP-qPCR using V5 antibody for empty vector (gray), WT-V5 (black), 3× RA V5-CBX8 (red) and W32A V5-CBX8 (blue) at *LMNB2* (C), *RUNX3* (D), *GATA6* (E) and *CCND2* (F), *n* = 3 biological replicates for all but W32A, which is *n* = 2. qPCR was performed in technical triplicate. For all qPCR, error bars represent SEM, *P*-values were calculated using two-tailed Student's *t*-test, *P < 0.05, **P < 0.01, ***P < 0.001, ****P < 0.0001.

To assess CBX8 binding at discrete genomic loci, we utilized Hs68 neonatal fibroblast cells, in which CBX8 binding sites have been identified and validated (42). Using ChIP-qPCR, we validated endogenous CBX8 enrichment at *GATA6, CCND2* and *RUNX3* but not at *LMNB2*, a constitutively expressed gene without CBX8 binding (Figure 6A). We used lentiviral transduction to make stable cell lines expressing an empty vector, V5-WT CBX8, 3× RA V5-CBX8 or W32A V5-CBX8 in the Hs68 cells, similarly as described for the T98Gs (Figure 6B). We performed chromatin immunoprecipitation followed by qPCR (ChIP-qPCR) with a V5 antibody to ensure immunoprecipitation of exogenously expressed CBX8. As with endogenous CBX8, we observe WT CBX8 enrichment at *GATA6, CCND2* and *RUNX3* but not *LMNB2* (Figure 6C–F). On the other hand, 3×RA and W32A CBX8 are not significantly enriched at these loci (Figure 6C–F). Together, these data suggest that both DNA and histone binding by the CD are critical for the association of CBX8 with chromatin, both globally and at discrete genomic sites. Further, to assess whether the binding decrease observed with the 3×RA and W32A mutants relates to a decrease in transcriptional repression, we examined gene expression changes upon expression of wild-type or mutant CBX8. We performed RNA-seq in the T98G CBX8 KO cell line with and without WT CBX8 re-expression and identified 423 genes that were significantly repressed upon CBX8 re-expression. We performed quantitative reverse transcription PCR (qRT-PCR) with five of the identified genes to examine expression changes with the 3×RA and W32A mutants. For most genes, re-expression of WT CBX8 repressed transcription, while one or both of the mutants failed to repress transcription (Supplementary Figure S7I). This suggests that DNA and methyl–lysine binding activity of the CBX8 CD are important for global chromatin binding, as well as proper transcriptional repression.

**Figure 7.**
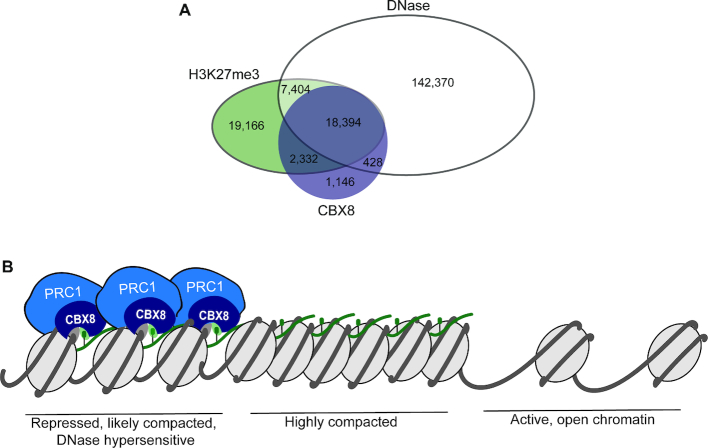
CBX8 and H3K27me3 associate with accessible DNA. (**A**) Overlap of DNase hypersensitive sites, (43,44) H3K27me3, (47) and CBX8 (47) in K562 cells. (**B**) Proposed model for the engagement of CBX8 with chromatin. PRC1 is shown in blue, with the CBX8 subunit in dark blue, engaging both H3K27me3 (green sphere) and DNA (gray). We propose that CBX8 will preferentially associate with DNase hypersensitive regions that are enriched in H3K27me3.

Our findings reveal that the CBX8 CD engages both DNA and H3K27me3 to drive chromatin association of CBX8. The high affinity with which it associates with DNA as compared to H3K27me3 implies that DNA accessibility could be a major factor in determining the subset of H3K27me3 regions bound by CBX8. In support of this model, analysis of genome-wide data from K562 cells revealed that 82% of CBX8 peaks correspond to regions of DNase hypersensitivity (43,44) (Figure 7A) and that the majority of the H3K27me3 peaks with DNase hypersensitivity are bound by CBX8 (Figure 7A). Based on these findings, the DNA binding ability of the CD may lead CBX8 to be selectively involved in chromatin compaction and gene silencing at genes with H3K27me3 in regions of accessible DNA, whereas the histone binding activity would act to retain it at H3K27me3 enriched regions (Figure 7B).

## DISCUSSION

Our findings in this study reveal a dual interaction mechanism for the CBX8 chromodomain, wherein engagement of both DNA and H3K27me3 mediate CBX8 association with chromatin. Importantly, we have shown that both binding modes are important for recovering full chromatin affinity *in vivo*. Our work bridges findings from two previous studies with conflicting conclusions regarding the importance of H3K27me3 for CBX8 chromatin association (17,56). Namely, bimolecular fluorescence complementation work suggested H3K27me3 is not necessary for chromatin association (56), while more recent kinetic studies conclude that H3K27me3 is important for CBX8 chromatin association (17). We find that H3K27me3 is indeed important for chromatin association, but is not entirely necessary, and that DNA binding contributes much of the binding energy of CD to nucleosomes. This is consistent with other studies, which have found that CBX proteins can bind nucleic acids, specifically RNA, via their chromodomains (16,57). In addition, several other CDs have been found to bind a variety of nucleic acid substrates (reviewed in (58)). Two main structural mechanisms for nucleic acid binding by CDs have been determined including the utilization of a highly basic αA helix (59–61) and interfaces that are partially overlapping with the methyl-lysine binding site (57,62). Notably, our data indicates a third structural mechanism of nucleic acid binding via an arginine-rich basic patch formed by the C-terminal portion of the β1 strand and αA helix.

There are two major implications from these results regarding the mechanism of reader domain mediated chromatin association. The first is the importance of DNA binding by canonical histone reader domains. Several studies have now demonstrated that histone reader domains, including chromodomains, bromodomains, PWWP domains, Tudor domains and PHD fingers, can interact with nucleic acids and that this binding is often much stronger than for histone peptides (58). Though the importance of this nucleic acid binding is not yet clear for all of these domains, our results indicate that one potential function is chromatin association independent of histone binding. This highlights that it is critical to study reader domain function in the context of the nucleosome.

The second major implication is that the local chromatin environment is likely critical in determining the contribution of reader domain binding to chromatin affinity. Despite the very weak affinity and moderate specificity for H3K27me3 to CBX8 CD *in vitro*, this binding interaction contributes significantly to the affinity of CBX8 for chromatin *in vivo*. This could be in part related to the increased effective concentration of repressive modifications in large regions of heterochromatin. For example HP1α is retained at H3K9me3-containing nucleosome arrays upon array compaction, due to both multivalent binding and probability of rebinding (63). This is similar to what is observed with bromodomains, which are often seen only to contribute substantially to chromatin affinity under conditions of hyper-acetylation (see Philpott *et al.*, for example) (64). CBX proteins may be similarly retained in large heterochromatin domains enriched in H3K27me3 *in vivo* (65). In addition, the weak specificity for H3K27me3 over H3K9me3 *in vitro* may be amplified by a combination of factors. For instance, HP1, which specifically associates with H3K9me3, will likely block CBX8 association this modification. In addition, as discussed above, a higher level of accessible DNA in H3K27me3 enriched regions as compared to H3K9me3 enriched regions could also bias CBX8 binding.

In *Drosophila*, the dPc CD interacts with H3K27me3 and has been shown to target the dPRC1 complex to facultative heterochromatin. In vertebrates, the five CBX paralogs bind H3K27me3 with drastically reduced affinity and specificity than the dPc CD, suggesting that this classical targeting mechanism may be more complex in vertebrates than in *Drosophila* (7,15). Additionally, our work highlights that these *in vitro* affinities of the CBX paralogs for H3K27me3 do not predict relative affinity for bulk chromatin *in vivo*, indicating that interactions outside of histone methylation drive chromatin association. We have identified that binding of the CBX8 CD to DNA is important for bulk chromatin association, which is likely also true for the other four CBX paralogs, as R20 and R22 are conserved across all five CBX paralogs and R19 and R60 are either R or K across all paralogs. In fact, the CBX2 CD has been shown to interact with DNA, although the structural basis for interaction and its role in chromatin association has not been assessed (66). Additional studies are necessary to fully understand the relative importance for DNA binding on CBX paralog association with chromatin.

CBX paralog expression is misregulated in countless cancers, and a myriad of other studies propose oncogenic mechanisms involving the CBX paralogs for numerous cancers. Further, a systematic structural analysis of methyl-lysine readers predicted the CBX chromodomains to be ‘druggable’ (67). As such, drug development initiatives have focused on developing paralog-specific CBX chromodomains inhibitors. To date, there has only been success in identifying CBX7 paralog-specific chromodomain inhibitors (68–70), which may be in part due to our limited knowledge of CBX binding mechanisms. Finally, these studies pave the way for the development of CBX8-specific chromodomain inhibitors, which will be useful tools to assess paralog-specific function and define the therapeutic utility of CBX8 inhibitors. While our work suggests that disruption of methyl binding may be sufficient to disrupt *in vivo* CBX8 binding, it also suggests that inhibitors that also disrupt DNA binding would be more potent.

## DATA AVAILABILITY

NMR data can be accessed on the Biological Magnetic Resonance Bank under accession number 27616 (BMRB, http://www.bmrb.wisc.edu/). RNA-seq data can be accessed on the NCBI GEO, accession number GSE123689. All data is available upon reasonable request.

## Supplementary Material

Supplementary Data
