# Supplementary material for: Engagement of DNA and H3K27me3 by the CBX8 chromodomain drives chromatin association
Source: Nucleic Acids Res. 2018 Dec 29;47(5):2289–305. doi: 10.1093/nar/gky1290 (PMC6411926; doi:10.1093/nar/gky1290)
Supplement: Supplementary Data [file gky1290_supplemental_files.pdf]

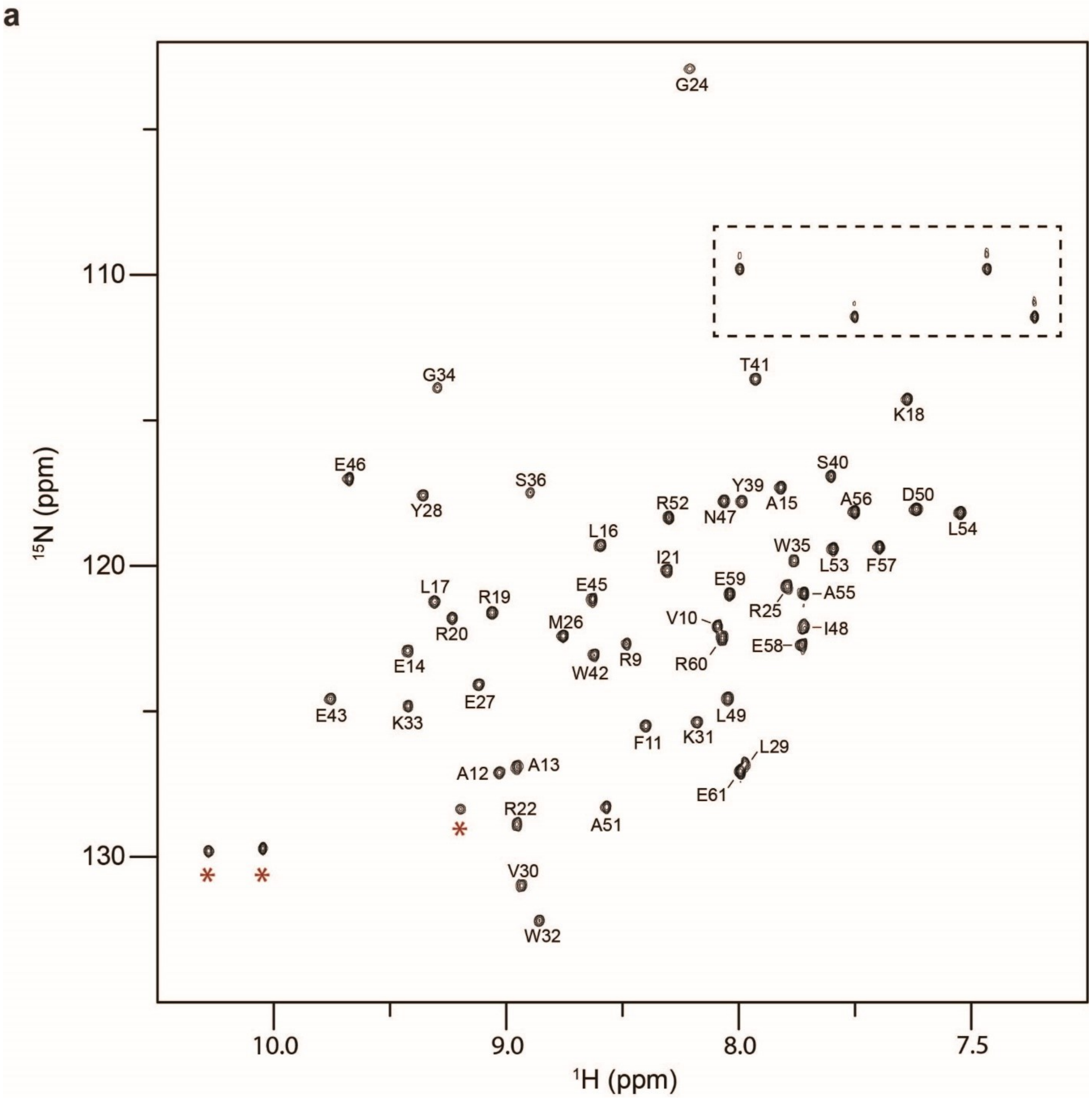

Supplementary Figure 1. a) ^{1}H-^{15}N-HSQC spectrum of the CD with assigned resonances labeled. Side chain NH peaks are enclosed in a dotted black square and tryptophan side chain resonances with a red star.

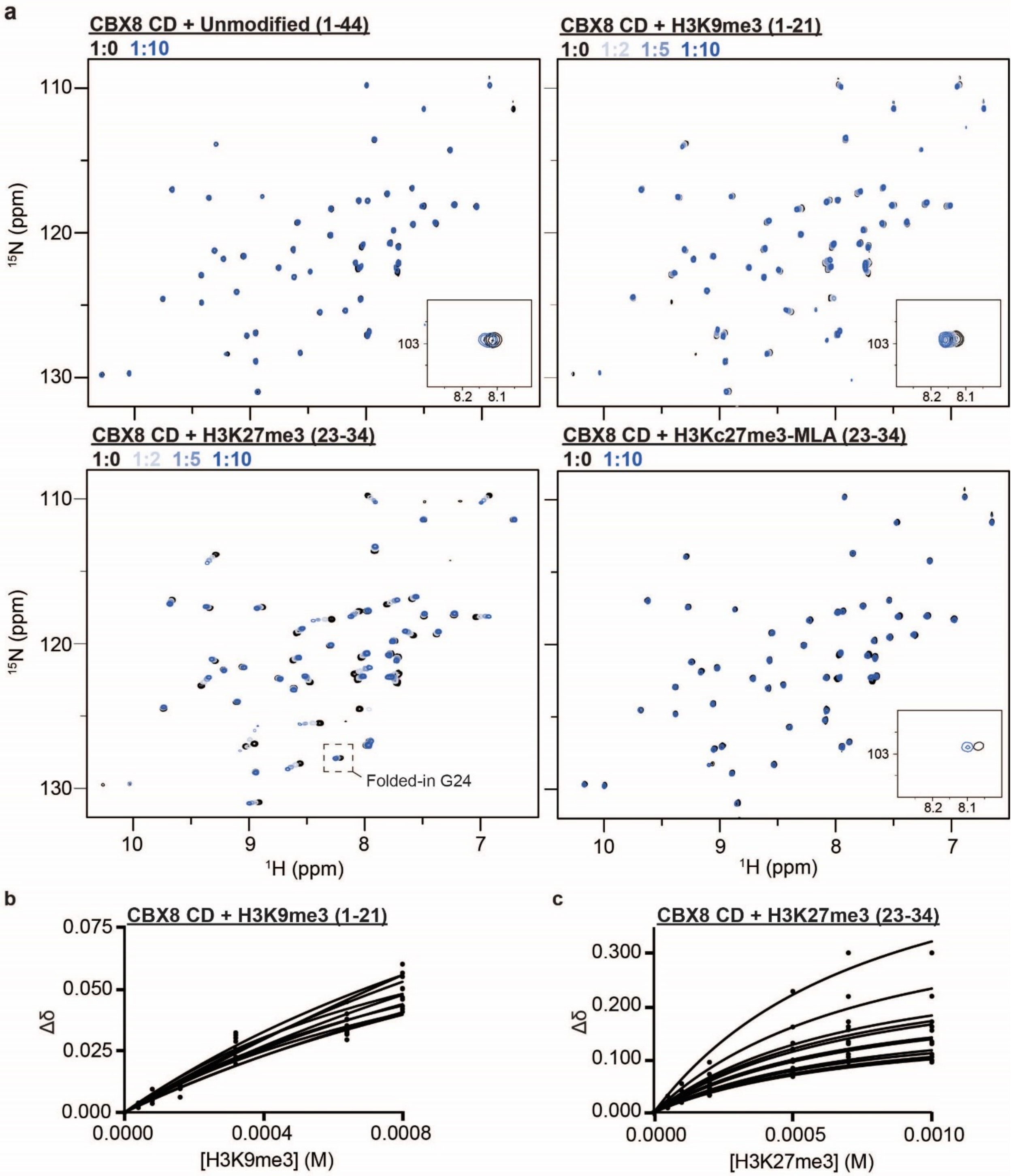

d

Peptide	Sequence	K_d (mM)
Unmodified (1-44)	ARTKQTARKSTGGKAPRKQLATKAARKSAPATGGVKKPHRYRPG	ND
H3K9me3 (1-21)	ARTKQTAR-K(Me3)-STGGKAPRKQLA	2.0 +/- 1.0
H3K27me3 (23-44)	KAAR-K(Me3)-SAPATGG	0.7 +/- 0.1

Supplementary Figure 2. a) Full 1H-15N-HSQC overlays for 15N-CD upon addition of increasing concentrations of unmodified H3 (1-44, top left), H3K9me3 (1-21, top right), H3K27me3 (23-34, bottom left) or H3Kc27me3 (23-34, bottom right) histone peptides. Glycine 24 is shown as an inset for clarity. b) Binding

curves for all resonances significantly perturbed in the H3K9me3 titration. c) Binding curves for all resonances significantly perturbed in the H3K27me3 titration. d) Table of determined K_d values and associated standard deviations for histone peptides tested. K_d values are reported as the average K_d value determined from analysis of the CSPs for all CD residues significantly perturbed. ND indicates binding not detected.

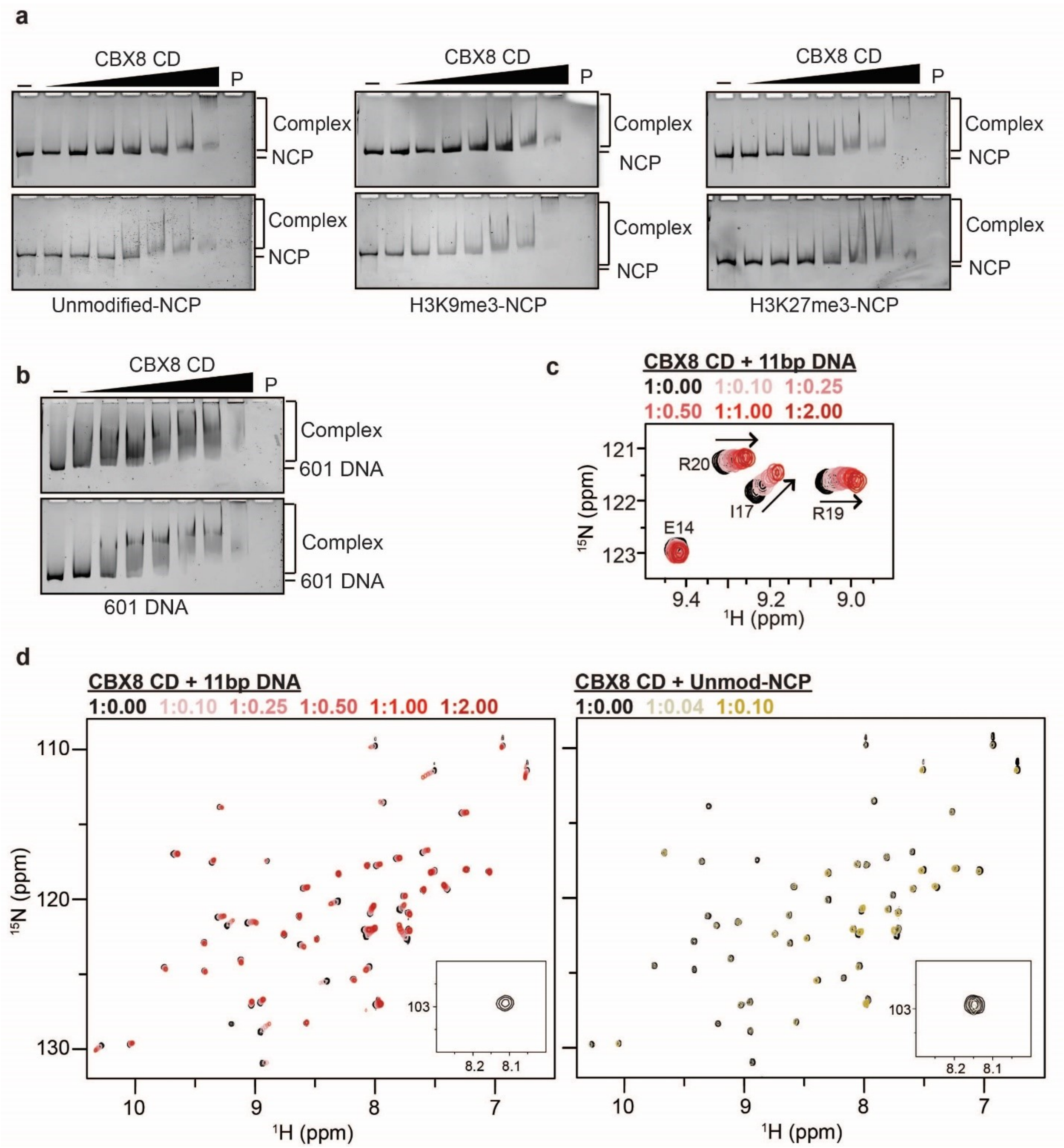

Supplementary Figure 3. a) EMSAs performed in triplicate with CD and unmodified (left), H3K9me3 (middle) and H3K27me3 (right) NCPs. b) EMSAs performed in triplicate with CD and the 147bp 601 DNA. c) ^{1}H-^{15}N-HSQC overlays for ^{15}N-CD upon addition of increasing concentrations of an 11bp DNA d) Full ^{1}H-^{15}N-HSQC overlays for ^{15}N-CD upon addition of increasing concentrations of the 11bp DNA (left) or unmodified NCP (right). Glycine 24 is shown as an inset for clarity.

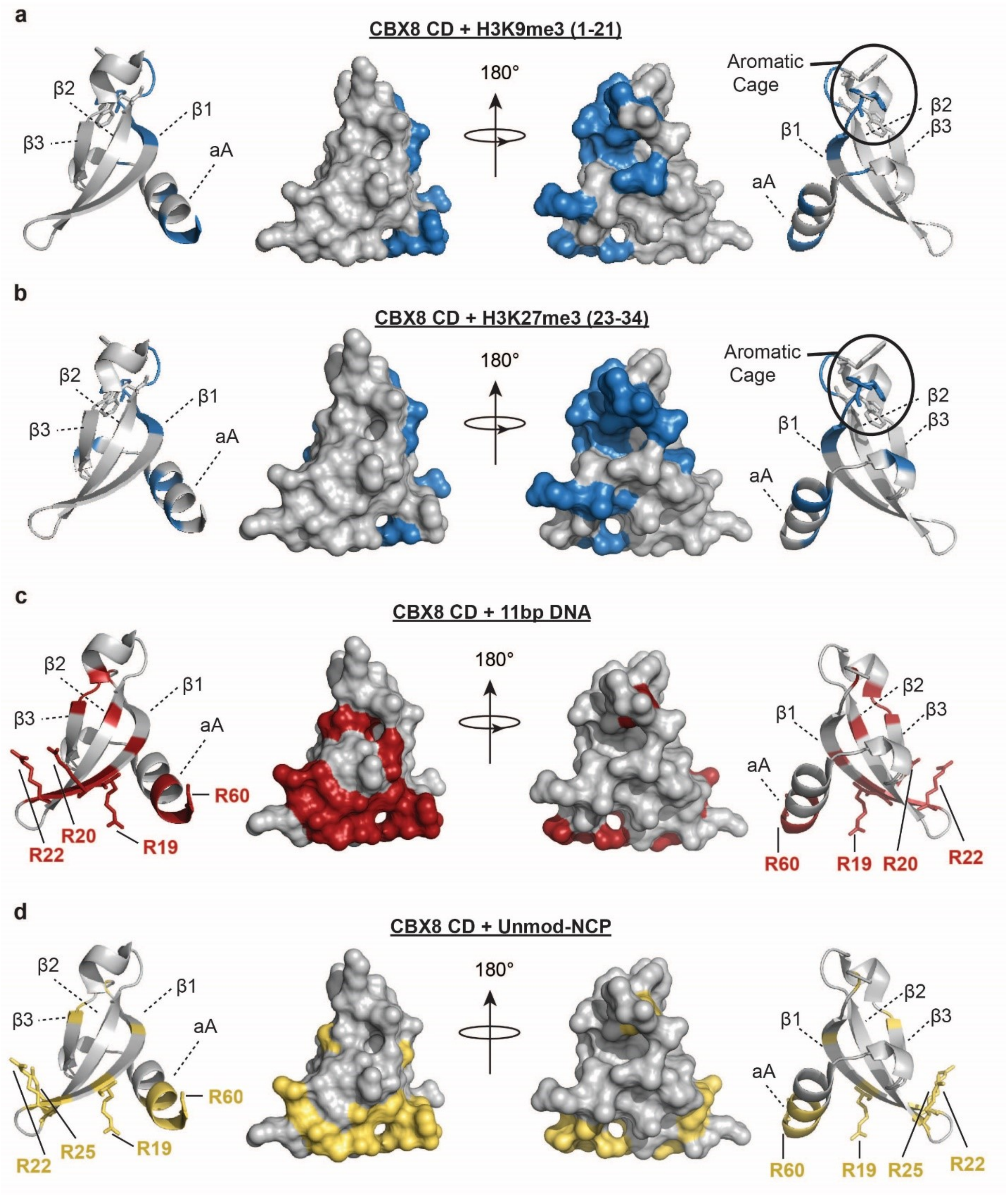

Supplementary Figure 4 a) Residues with significant CSPs upon addition of H3K9me3 peptide plotted onto a cartoon and surface representation of the CD (PDBID 3I91) and colored blue. Two orientations of the structure are shown rotated 180° about the y-axis. b) Residues with significant CSPs upon addition of H3K27me3 peptide plotted onto a cartoon and surface representation of the CD (PDBID 3I91) and colored blue. Two orientations of the structure are shown rotated 180° about the y-axis. c) Residues with significant CSPs upon addition of 11bp DNA plotted onto a cartoon and surface representation of the CD (PDBID

3I91) and colored red. Two orientations of the structure are shown rotated 180° about the y-axis. d) Residues with significant CSPs upon addition of unmodified NCP plotted onto a cartoon and surface representation of the CD (PDBID 3I91) and colored gold. Two orientations of the structure are shown rotated 180° about the y-axis.

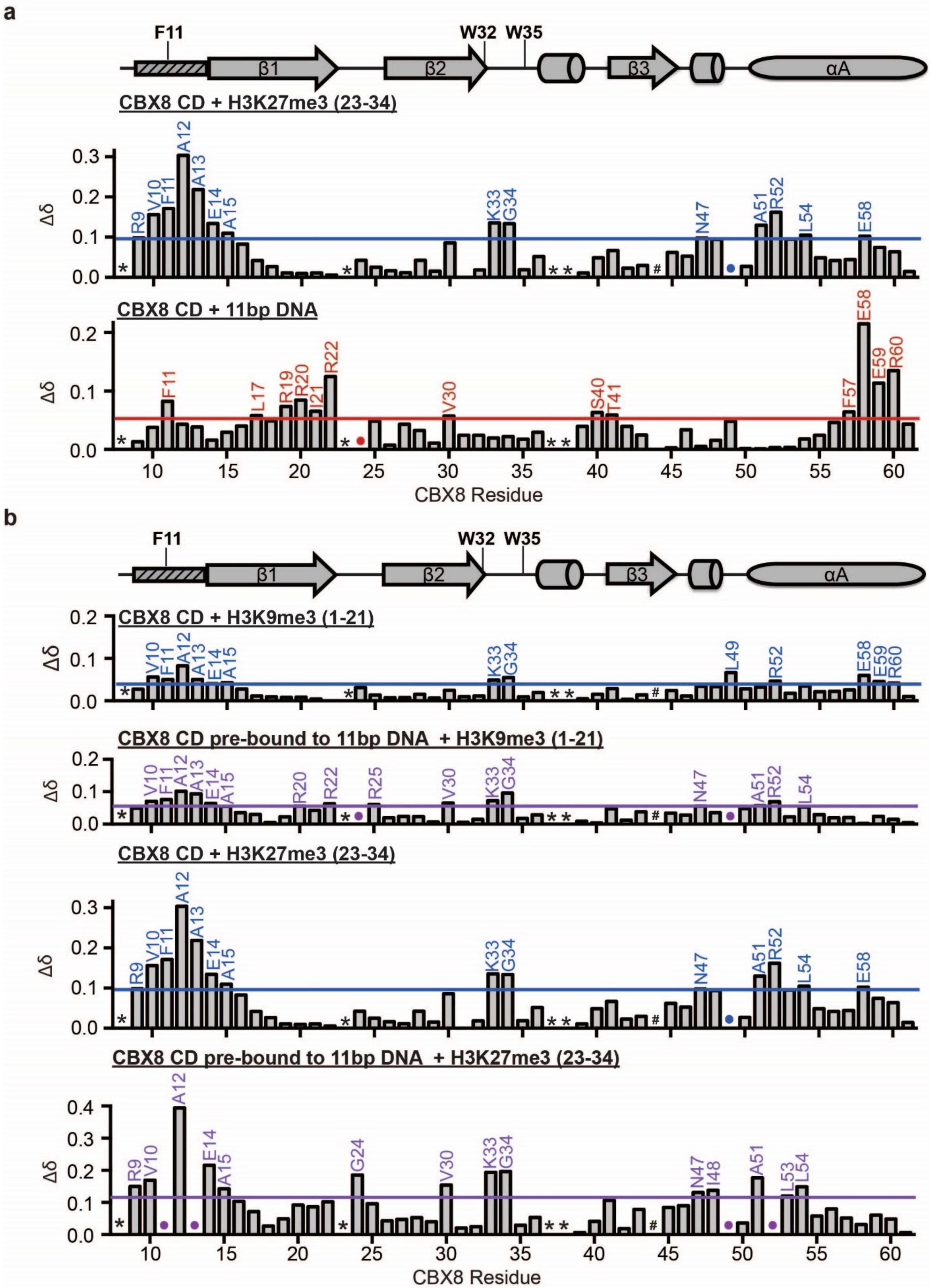
a
F11
W32
W35
β1
β2
β3
αA
CBX8 CD + H3K27me3 (23-34)
Δδ
R9
V10
F11
A12
A13
E14
A15
K33
G34
N47
A51
R52
L54
E58
CBX8 CD + 11bp DNA
F11
L17
R19
R20
I21
R22
V30
S40
T41
F57
E58
E59
R60
CBX8 Residue
b
CBX8 CD + H3K9me3 (1-21)
L49
CBX8 CD pre-bound to 11bp DNA + H3K9me3 (1-21)
R25
CBX8 CD + H3K27me3 (23-34)
CBX8 CD pre-bound to 11bp DNA + H3K27me3 (23-34)
G24
I48
L53
CBX8 Residue

Supplementary Figure 5. a) Normalized CSP (Δδ) between the apo, H3K27me3-bound (1:10 ratio, top) and DNA-bound (1:2.00 ratio, bottom) spectra are plotted against CBX8 residue number. b) Normalized CSP (Δδ) between the apo and H3K9me3-bound (1:10 ratio, top), DNA and H3K9me3-bound (1:1:10, middle-top), apo and H3K27me3-bound (1:10, middle-bottom) and DNA and H3K27me3-bound (1:1:10, bottom) spectra are plotted against CBX8 residue number. For (a) and (b) the secondary structure of CD from the crystal structure PDBID 3I91 is diagramed above the Δδ plots with the aromatic cage residues labeled. The small rectangle with dashed lines represents the region of CD that undergoes a conformational change between apo and histone bound states in the crystal structure. * indicates missing resonances, # indicates proline residue and colored dots represent resonances that broaden beyond detection during the experiment

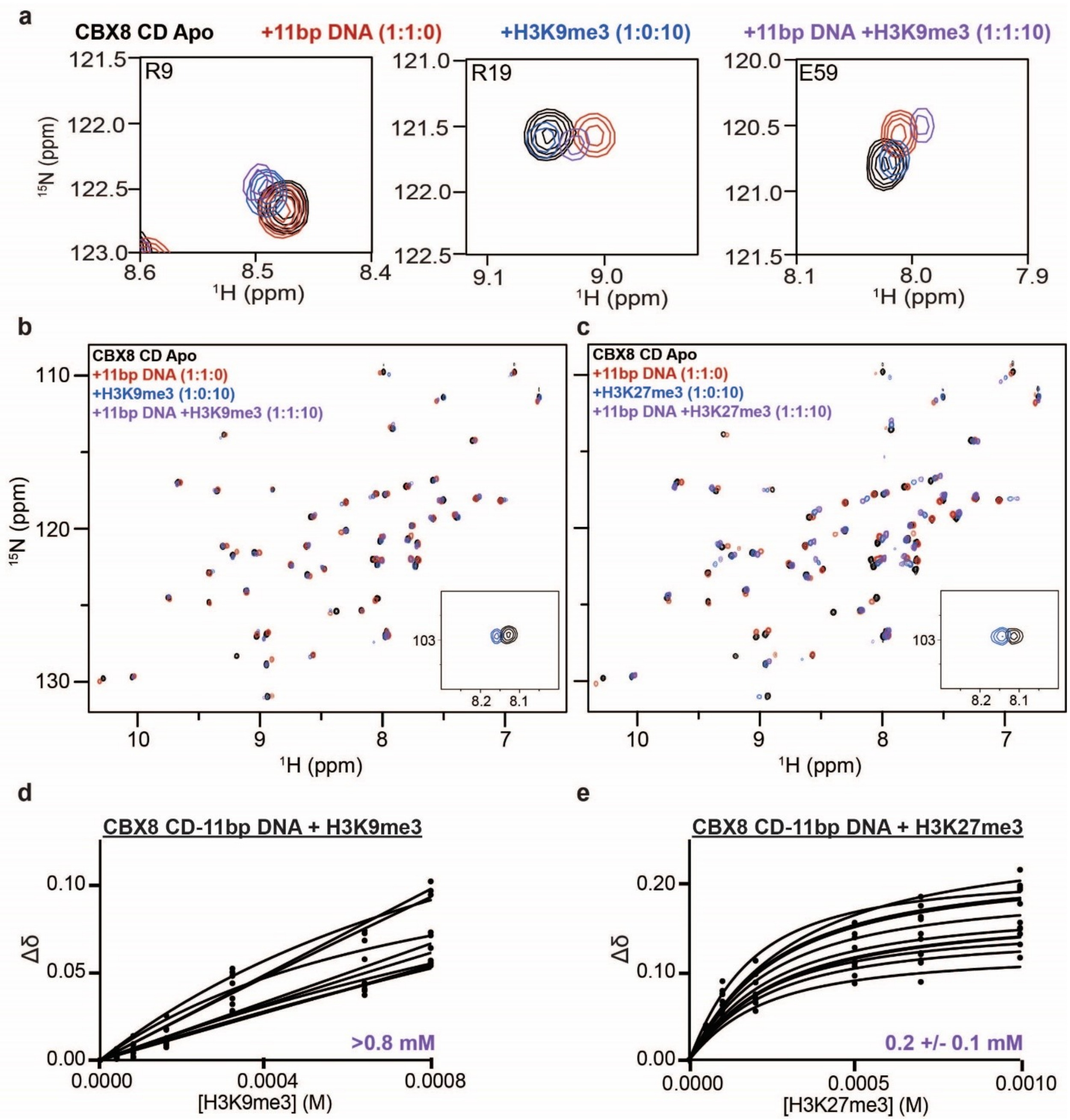

Supplementary Figure 6. a) ^{1}H-^{15}N-HSQC overlays for ^{15}N-CD in the apo (black, 1:0:0 ratio), bound to an 11bp DNA (red, 1:1:0 ratio), bound to H3K9me3 (blue, 1:0:10), or bound to both 11bp DNA and H3K9me3 (purple, 1:1:10 ratio). Shown are resonances for selected residues in the histone binding pocket (R9, left), DNA binding pocket (R19, middle) and a residue sensitive to both DNA and histone binding (E59, right) are shown. b) Full ^{1}H-^{15}N-HSQC overlays for ^{15}N-CD in the apo (black, 1:0:0 ratio), bound to an 11bp DNA (red, 1:1:0 ratio), bound to H3K9me3 (blue, 1:0:10), or bound to both 11bp DNA and H3K9me3 (purple, 1:1:10 ratio). to H3K27me3 (blue, 1:0:10), or bound to both 11bp DNA and H3K27me3 (purple, 1:1:10 ratio). Glycine 24 is shown as an inset for clarity. c) Full ^{1}H-^{15}N-HSQC overlays for ^{15}N-CD in the apo (black, 1:0:0 ratio), bound to an 11bp DNA (red, 1:1:0 ratio), bound to H3K27me3 (blue, 1:0:10), or bound to both 11bp DNA and H3K27me3 (purple, 1:1:10 ratio). Glycine 24 is shown as an inset for clarity. d) Binding curves for all resonances significantly perturbed in the H3K9me3 titration with the CD pre-bound to 11bp DNA. e) Binding curves for all resonances significantly perturbed in the H3K27me3 titration with the CD pre-bound to 11bp DNA.

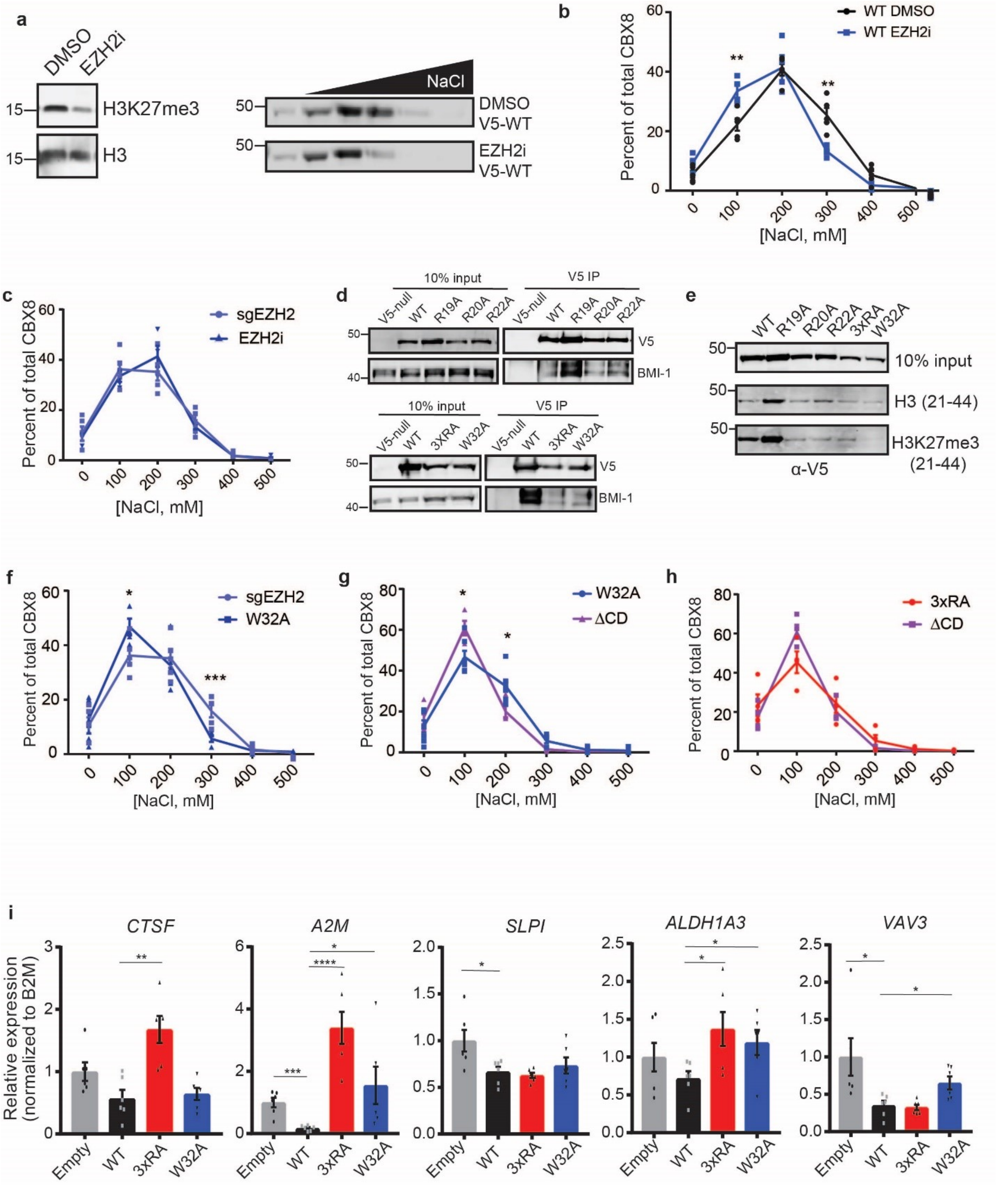

Supplementary Figure 7. a) Immunoblot of H3K27me3 levels following 48 h EZH2i treatment (left), immunoblot of a representative SSE of V5-WT + EZH2i (anti-V5) (right). b) Quantitation of the SSEs from a) to compare CBX8 elution between EZH2i (blue) and DMSO (black) treated cells, n=4, 5 biological replicates, respectively. c) Quantitative comparison of EZH2i SSE (dark blue) and sgEZH2 SSE (light blue) shown previously, n= 4 ,5 biological replicates respectively. d) Co-immunoprecipitation of V5 mutants using anti-V5. Immunoblots were stained for V5 and the PRC1 subunit BMI-1. e) Peptide pulldowns of all mutants

with unmodified H3 and H3K27me3 (anti-V5). f) Quantitative comparison of sgEZH2 (light blue) and W32A SSE (dark blue) quantitation n=5, 4 biological replicates, respectively. g) Quantitative comparison of W32A (blue) and ΔCD SSE (purple) previously shown, n=7, 4 biological replicates respectively. h) Quantitative comparison of 3xRA (red) and ΔCD SSEs (purple) shown previously, n=4 biological replicates for both. i) qRT-PCR in T98G KO, WT, 3xRA, and W32A cell lines, n= 6 biological replicates, performed in technical triplicate. For all SSE quantitations, the amount of CBX8 in each fraction is reported as a percentage of total CBX8. Error bars are the SEM. Two-tailed student's t-tests were used to determine significance; * $p<0.05$, ** $p< 0.01$, *** $p< 0.001$, **** $p< 0.0001$
